# Biochemical Basis of Xylooligosaccharide Utilisation by Gut Bacteria

**DOI:** 10.3390/ijms23062992

**Published:** 2022-03-10

**Authors:** Ravindra Pal Singh, Raja Bhaiyya, Raksha Thakur, Jayashree Niharika, Chandrajeet Singh, Dimitrios Latousakis, Gerhard Saalbach, Sergey A. Nepogodiev, Praveen Singh, Sukesh Chander Sharma, Shantanu Sengupta, Nathalie Juge, Robert A. Field

**Affiliations:** 1 Division of Food and Nutritional Biotechnology, National Agri-Food Biotechnology Institute (NABI), SAS Nagar 140306, India; raja.bhaiyya@nabi.res.in (R.B.); raksha.th@nabi.res.in (R.T.); jayashree.p@nabi.res.in (J.N.); chndrjtsngh@gmail.com (C.S.); 2 Department of Biological Chemistry, John Innes Centre, Norwich Research Park, Norwich NR47UH, UK; gerhard.saalbach@jic.ac.uk (G.S.); sergey.nepogodiev@jic.ac.uk (S.A.N.); 3 The Gut Microbes and Health Institute Strategic Programme, Quadram Institute Bioscience, Norwich Research Park, Norwich NR4 7UQ, UK; dimitris.latousakis@quadram.ac.uk (D.L.); nathalie.juge@quadram.ac.uk (N.J.); 4 CSIR-Institute of Genomics and Integrative Biology, Mathura Road, New Delhi 110025, India; praveen05singh@gmail.com (P.S.); shantanus@igib.res.in (S.S.); 5 Department of Biochemistry, South Campus, Panjab University, Chandigarh 160014, India; sukeshcs@pu.ac.in

**Keywords:** xylan, *Limosilactobacillus reuteri*, *Blautia producta*, glycoside hydrolase, β-xylosidases, xylooligosaccharide, gut microbiota, carbohydrate-active enzymes

## Abstract

Xylan is one of the major structural components of the plant cell wall. Xylan present in the human diet reaches the large intestine undigested and becomes a substrate to species of the gut microbiota. Here, we characterised the capacity of *Limosilactobacillus reuteri* and *Blautia producta* strains to utilise xylan derivatives. We showed that *L. reuteri* ATCC 53608 and *B. producta* ATCC 27340 produced β-D-xylosidases, enabling growth on xylooligosaccharide (XOS). The recombinant enzymes were highly active on artificial (*p*-nitrophenyl β-D-xylopyranoside) and natural (xylobiose, xylotriose, and xylotetraose) substrates, and showed transxylosylation activity and tolerance to xylose inhibition. The enzymes belong to glycoside hydrolase family 120 with Asp as nucleophile and Glu as proton donor, as shown by homology modelling and confirmed by site-directed mutagenesis. In silico analysis revealed that these enzymes were part of a gene cluster in *L. reuteri* but not in *Blautia* strains, and quantitative proteomics identified other enzymes and transporters involved in *B. producta* XOS utilisation. Based on these findings, we proposed a model for an XOS metabolism pathway in *L. reuteri* and *B. producta* strains. Together with phylogenetic analyses, the data also revealed the extended xylanolytic potential of the gut microbiota.

## 1. Introduction

The human gut harbours about 100 trillion microbes constituting the human gut microbiota (HGM) [1,2]. The HGM performs a wide variety of functions vital for normal physiology, including the development of the gastrointestinal tract [3], promoting maturation of the immune system [4], pathogen exclusion [5,6], and improving energy capture from dietary components through fermentation [7]. The importance of the HGM in human health has been underpinned by large metagenome sequencing programmes that revealed an enormous catalogue of microbial genes [2,8,9]. Nevertheless, the huge amount of sequencing data has far exceeded our ability to accurately annotate and predict the function of those genes. Indeed, it is estimated that about 75% of genes encoded by the HGM remain to be functionally characterised, which may lead to the discovery of novel protein families [10].

Dietary glycans are recalcitrant to digestion by human-encoded enzymes, thus the major portion of polysaccharides reaches the large intestine, where they provide a nutrient source for symbiotic communities of HGM [11]. The HGM is dominated by bacterial phyla belonging to *Actinobacteria, Bacteroidetes*, *Proteobacteria,* and *Firmicutes* [2]. Together these bacterial species can metabolise various complex glycans present in our diets such as pectin, xylan, and resistant starch [12]. These glycans are structurally complex due to various degrees of polymerisation (DP), branching patterns with different glycosidic linkages, and monosaccharide composition [13]. The enzymes involved in the breakdown of complex glycans belong to the carbohydrate active enzymes (CAZymes) and are categorised into glycoside hydrolases (GHs), esterases, and polysaccharide lyases (PLs) [14,15,16] (www.cazy.org (accessed on 1 January 2022)).

Xylan is the most abundant and complex type of glycans present in cell walls of various cereal grains consumed by humans; for example, wheat, rye, and oat [17]. It consists mainly of a linear backbone of β-1,4-D-xylopyranoside units that are often modified with 4-O-methyl-glucuronyl, acetyl, feruloyl, and α-L-arabinofuranosyl residues. Xylan can be categorised into homoxylan and heteroxylan based on substituted groups, which include glucuronoxylan (GX), arabinoxylan (AX), and glucuronoarabionoxylan (GAX) [18]. *Bacteroides* and *Roseburia* species have been shown to produce endoxylanases for breaking down the backbone of this complex polysaccharide [19,20]. In *Bacteroides*, the xylan degradation machinery is encoded by a polysaccharide utilisation locus, encompassing outer membrane enzymes that cleave long glycan chains into oligosaccharides [21]. The latter are then imported into the periplasm by TonB-dependent transporters, where they are further degraded into shorter oligosaccharides and then monosaccharides, as shown for *B. intestinalis* DSM 17393 [22]. In contrast, the utilisation of arabinoxylans in *Roseburia intestinalis* L1-82, a member of the Firmicutes phylum, is through multimodular cell-attached xylanases that digest complex glycans into oligosaccharides, which are imported into the cytoplasm by an ATP-binding cassette (ABC) transporter [23]. However, many Gram-positive bacteria lack such glycan-utilising repertoires, and therefore rely on the presence of primary consumers of xylan, such as *Bacteroides ovatus* ATCC8483, to provide xylan degradation products as a nutrient source through cross-feeding [24].

Xylan breakdown products such as arabinoxylan-oligosaccharides (AXOS) and xylooligosaccharides (XOS) have prebiotics effects through the modulation of the HGM composition, especially the populations of *Bifidobacteria*, *Lactobacilli*, *Bacteroides,* and *Prevotella* [25]. For example, the production of XOS by *Bacteroidetes* has been shown to promote the expansion of population of *Bifidobacterium animalis* subsp. *lactis* DSM-10140 in the gut through xylose cross-feeding [26]. *Bifidobacterium* are known to show cooperative behaviour for thriving on AXOS and XOS [27,28]. Here, we focused on XOS utilisation by *Limosilactobacillus reuteri* and *Blautia producta*, two beneficial Gram-positive bacterial symbionts found in a wide range of vertebrates [29,30,31,32]. *L. reuteri* strains have a long history of beneficial dietary effects and are associated with a wide range of animal hosts [29,30,33,34,35]. In contrast, the potential benefits of *B. producta* have only recently begun to be appreciated [31,32], and it is now considered a beneficial genus with potential probiotic properties [36]. Despite their beneficial association with host, the dietary glycan-utilising potential of *B. producta* strains remains to be determined.

## 2. Results and Discussion

### 2.1. L. reuteri and B. producta Strains Utilise XOS as Metabolic Substrate

**Figure 1:**
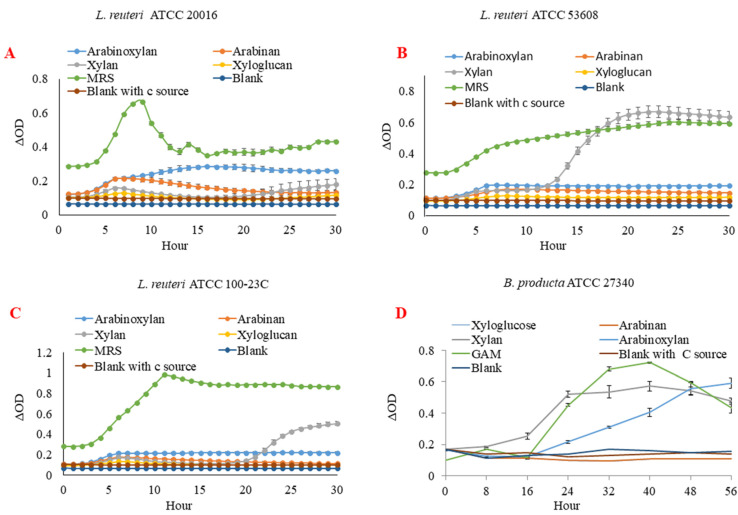
Growth curves of *L. reuteri* and *B. producta* strains on different carbon sources. *L. reuteri* strains ATCC 20016 (**A**), ATCC 53608 (**B**), ATCC 100-23C (**C**), and *B. producta* ATCC 27340 (**D**) were grown on arabinan, arabinoxylan, xylan (xylooligosaccharides), and xyloglucan. Blank—minimal medium with bacterium inoculated but no additional carbon source. Blank with c source—minimal medium with xylan as a main carbon source. MRS - de Man, Rogosa, and Sharpe medium. GAM- Gifu anaerobic medium.

*L. reuteri* ATCC 100-23C, *L. reuteri* ATCC 53608, *L. reuteri* ATCC 20016, and *B. producta* ATCC 27340 strains were tested on a range of substrates, including xylan (XOS), arabinoxylan, arabinan, and xyloglucan. *B. producta* ATCC 27340 was the only strain showing growth on arabinoxylan, whereas all *L. reuteri* strains could grow on XOS. None of the strains could grow on arabinan and xyloglucan (Figure 1). The structural characterisation of XOS showed that it consisted of up to DP 10 as determined by MALDI-MS and HPAEC-PAD (Figure S1), and it was not acetylated (Figure S2B,C). This analysis was in alignment with previously characterised XOS [37].

The capacity of *L. reuteri* and *B. producta* strains to utilise XOS was then determined by analysing the supernatant of the strains by HPAEC-PAD and HPAEC-ED, respectively. The digestion of XOS was evident for *L. reuteri* ATCC 100-23C, *L. reuteri* ATCC 53608, and *B. producta* ATCC 27340, with the appearance of a peak corresponding to xylose within 24 h of growth that continued to increase until 48 h (Figures S3 and S4). Furthermore, the HPAEC chromatograms clearly indicated that XOS up to DP 4 was utilised by only three strains (except *L. reuteri* ATCC 20016) within 72 h of incubation.

**Figure 2:**
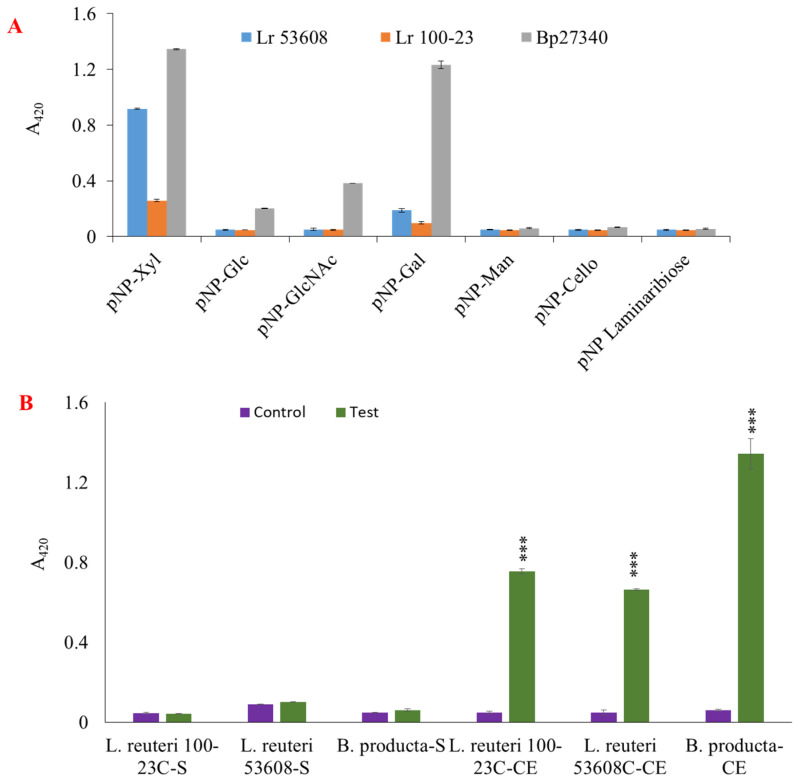
Enzymatic activity of *L. reuteri* and *B. producta* strains grown on XOS. The enzymatic activity of bacterial cell lysates from *L. reuteri* ATCC 53608 (Lr 53608), *L. reuteri* ATCC 100-23C (Lr 100-23), and *B. producta* ATCC 27340 (Bp27340) was determined using the artificial substrates *p*NP-Xyl, *p*NP-Glc, *p*NP-Cello, *p*NP-GlcNAc, *p*NP-Gal, *p*NP-Man, and *p*NP-laminaribiose (**A**). Cell extract (CE) and bacterial supernatant (S) were used to determine enzymatic activity using *p*NP-Xyl (**B**) as a substrate (2 mM final concentration). The results are representative of three replicates, and differences between control and test were analyzed by two-way ANOVA and Bonferroni post-tests (*** *p* = 0.001).

Next, we assessed the ability of the strains to degrade artificial substrates by monitoring the activity of the cell lysates and supernatants of the strains grown on XOS on *p*NP-laminaribiose, *p*NP-Cello, *p*NP-Mann, *p*NP-Xyl, *p*NP-Glc, *p*NP-Gal, and *p*NP-GlcNAc. *B. producta* ATCC 27340 was shown to have a broad substrate specificity against all artificial substrates tested, whilst *L. reuteri* ATCC 100-23C and *L. reuteri* ATCC 53608 showed enzymatic activity against *p*NP-Xyl and *p*NP-Gal (Figure 2A). The xylanolytic activity on these artificial substrates was mainly present in the cell lysate fractions of *L. reuteri* ATCC 100-23C, *L. reuteri* ATCC 53608, and *B. producta* ATCC 27340 as compared to culture supernatants (Figure 2B).

### 2.2. L. reuteri and B. Producta β-xylosidases Are Involved in XOS Utilisation

**Figure 3:**
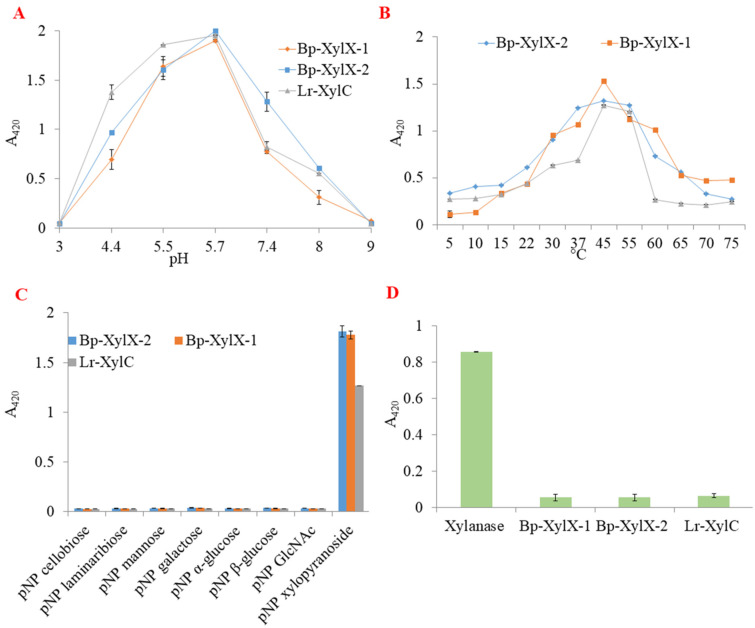
Characterisation of Bp-XylX-1, Bp-XylX-2, and Lr-XylC β-xylosidase enzymatic activity. The optimum (**A**) pH and (**B**) temperature of recombinant β-xylosidases were determined using *p*NP-Xyl (2 mM final concentration) in different buffers and temperatures. (**C**) The enzymatic activity of recombinant β-xylosidases was determined on artificial substrates at 2 mM final concentration. (**D**) Assessment of endolytic activity was performed with K-Xly 6-*p*NP (2 mM final concentration) where a commercial xylanase was used as control.

Genome analysis identified two predicted β-xylosidases (gene IDs 2515951826 and 2515951164) in the *B. producta* ATCC 27340 genome, one (gene ID 2843021658) in *L. reuteri* ATCC 53608, and one (gene ID 2500071404) in *L. reuteri* ATCC 100-23C. No predicted β-xylosidase gene could be identified in the genome of the *L. reuteri* ATCC 20016 strain. In order to characterise the enzymes involved in XOS utilisation, the putative β-xylosidases from *B. producta* ATCC 27340, Bp-XylX-1, Bp-XylX-2, and *L. reuteri* ATCC 53608, Lr-XylC were heterologously produced in *E. coli*. The β-xylosidase from *L. reuteri* ATCC 53608 was chosen for future characterisation based on the growth of this strain on XOS as compared to *L. reuteri* ATCC 100-23C. The purified recombinant proteins showed an apparent size of ~75 kDa on 10% SDS-PAGE, in accordance with their theoretical MW (Figure S5A). All three enzymes were highly active on *p*NP-Xyl in the range of pH between 5.5–5.7 using citrate and sodium phosphate buffers, while the enzymatic activity was drastically decreased in Tris-HCl buffer (Figure 3A). The recombinant enzymes showed a broad temperature tolerance on this substrate, with an optimum between 37 °C and 55 °C (Figure 3B).

The purified enzymes were again tested on the artificial substrates *p*NP-laminaribiose, *p*NP-Cello, *p*NP-Mann, *p*NP-Xyl, *p*NP-Glc, *p*NP-Gal, and *p*NP-GlcNAc, revealing the strict specificity of the three enzymes for *p*NP-Xyl (Figure 3C). No enzymatic activity was observed on K-Xly 6-*p*NP (Figure 3D). Bp-XylX-1, Bp-XylX-2, and Lr-XylC shared similar *K*_m_ values on *p*NP-Xyl (0.19 ± 0.02 mM, 0.19 ± 0.02 mM, 0.23 ± 0.0 mM, respectively), but *k*_cat_ was slightly lower for Bp-XylX-2 (10.7 ± 0.2 s^−1^) as compared to Bp-XylX 1 (11.9 ± 0.23 s^−1^) and Lr-XylC (11.4 ± 0.2 s^−1^). The highest catalytic efficiency was observed for Bp-XylX-1, at 63 s^−1^ mM^−1^ (Figure S5B and Table 1). The kinetics parameters of these β-xylosidases were better than other β-xylosidases from gut bacteria characterised to date on pNP-Xyl, such as *Bacteroides ovatus* ATCC 8483 (with K_m_ and k_cat_/K_m_ of 1.71 mM and 2.68 ± 0.08 s^−1^mM^−1^, respectively) [38], *Bifidobacterium breve* K-110 (with a K_m_ of 0.87 mM) [39], or *Bifidobacterium animalis* subsp. *lactis* BB-12 (with K_m_ and k_cat_ of 15.6 ± 4.2 mM and 60.6 ± 10.8 s^−1^, respectively) [40].

**Figure 4:**
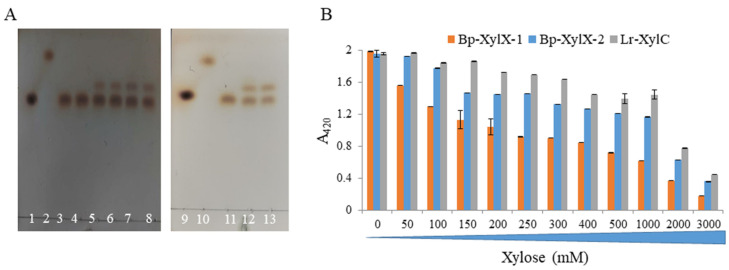
Transxylosylation activity and xylose tolerance capability of Bp-XylX-1, Bp-XylX-2, and Lr-XylC. Transxylosylation activity was performed using ethanol and methanol as donors and *p*NP-xyl as a receptor (**A**). 1. xylose; 2. *p*NP-xyl; 3. *p*NP-xyl + Bp-XylX-1; 4. *p*NP-xyl + Bp-XylX-2; 5. *p*NP-xyl + Bp-XylX-1 + methanol; 6. *p*NP-xyl + Bp-XylX-1 + ethanol; 7. *p*NP-xyl + Bp-XylX-2 + methanol; 8. *p*NP-xyl + Bp-XylX-2 + ethanol; 9. xylose; 10. *p*NP-xyl; 11. *p*NP-xyl + Lr-XylC; 12. *p*NP-xyl + Lr-XylC + methanol; 13. *p*NP-xyl + Lr-XylC + ethanol. Xylose inhibition assay was performed in the presence of *p*NP-xyl at 2 mM final concentration and various concentrations of xylose (**B**).

Transxylosylation activities of β-D-xylosidases were previously reported for enzymes from *Bacillus halodurans* [41], *Aspergillus* sp. [42,43], and *T. saccharolyticum* JW/SL-YS485 [44]. Here, we showed that Bp-XylX-1, Bp-XylX-2, and Lr-XylC released xylose from *p*NP-xylose as shown by TLC (Figure 4A), and showed transxylosylation activities when ethanol and methanol were used as donors. The transfer of xylose released from *p*NP-xylose to ethanol or methanol resulted in the formation of alkyl alcohols as confirmed by TLC (Figure 4A). The formation of ethoxylated (A) and methoxylated (B) xyloses was further confirmed by ESI-MS, showing the presence of 201 m/z (M + Na^+^) and 187 m/z (M + Na^+^), respectively (Figure S6).

Interestingly, all three β-xylosidases showed high tolerance to inhibition by xylose, with only ~42%, ~26%, and ~12% reduction in activity determined in the presence of 200 mM xylose concentration for Bp-XylX-1, Bp-XylX-2, and Lr-XylC, respectively (Figure 4B). Lr-XylC showed highest tolerance to xylose concentration, with only ~26% reduction in activity up to 1 M xylose, while the activity was drastically reduced for all three β-xylosidases when the concentration of xylose was further increased (Figure 4B). Such tolerance for xylose was previously reported for a β-xylosidase of the *Massilia* species, isolated from the faeces of *Rhinopithecus bieti*, revealing ~50% inhibition in activity in the presence of 500 mM of xylose concentration [45], while a highly tolerant β-xylosidase from *Dictyoglomus thermophilum* DSM 3960 (isolated from a hot spring) showed only 40% inhibition in the presence of 3 M xylose [46]. In contrast, β-xylosidases of fungal origin are known to tolerate low concentrations of xylose. For example, the β-xylosidase from *Scytalidium thermophilum* was insensitive to xylose inhibition up to 200 mM xylose (highest value tested) [47] while Ts-XOS showed 30% inhibition at 200 mM xylose [44]. In addition, β-xylosidases from *Humicolagrisea* var*. thermoidea* [48] and *Aspergillus nidulans* [49] were inhibited by a low concentration of xylose, with the latter showing 44% inhibition by 25 mM xylose. The β-xylosidases (Bp-XylX-1, Bp-XylX-2, and Lr-XylC) characterised in this work showed high tolerance to inhibition by xylose, which may be valuable for biotechnological applications.

**Figure 5:**
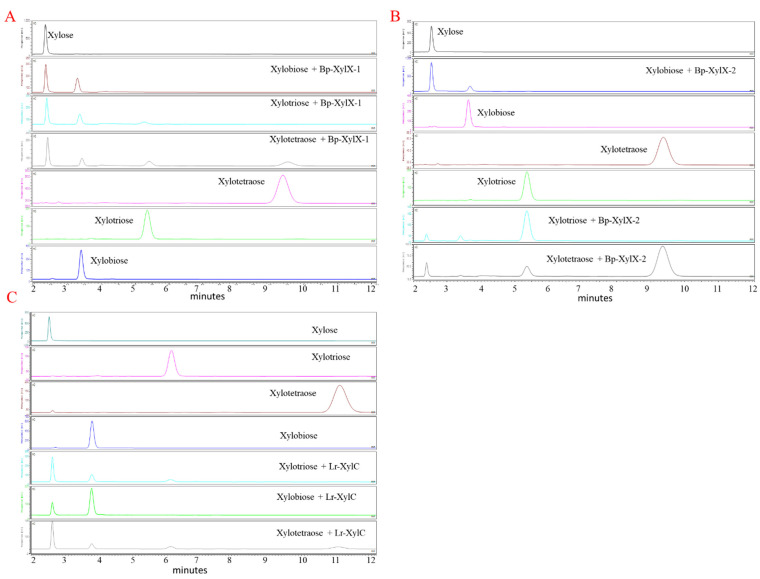
Enzymatic activity of Bp-XylX-1, Bp-XylX-2, and Lr-XylC β-xylosidases on xylobiose, xylotriose, and xylotetraose. The reaction was carried out at 37 °C for 15 min, and the products of digestion by Bp-XylX-1 (**A**), Bp-XylX-2 (**B**), and Lr-XylC (**C**) were determined by HPAEC-ED. An isocratic gradient of 200 mM NaOH was used in A and B, while 150 mM was used for C.

Bp-XylX-1, Bp-XylX-2, and Lr-XylC shared similar degradation patterns when xylobiose, xylotriose, and xylotetraose were used as substrates with release of xylose after 15 min as determined by HPAEC-ED (Figure 5A–C) and FACE (Figure S7). Furthermore, these digestion profiles suggested that Bp-XylX-1 and Lr-XylC (Figure 5A,C) could access longer-chain XOS than Bp-XylX-2 (Figure 5B), as significant amounts of xylotriose and xylotetraose remained in latter’s enzymatic degradation profiles (Figure 5). HPAEC-ED confirmed the exo-acting activity of the recombinant β-xylosidases, where xylotriose and xylotetraose were first converted into xylobiose and xylotriose, and then xylose and xylobiose, respectively (Figures S8–S10).

**Figure 6:**
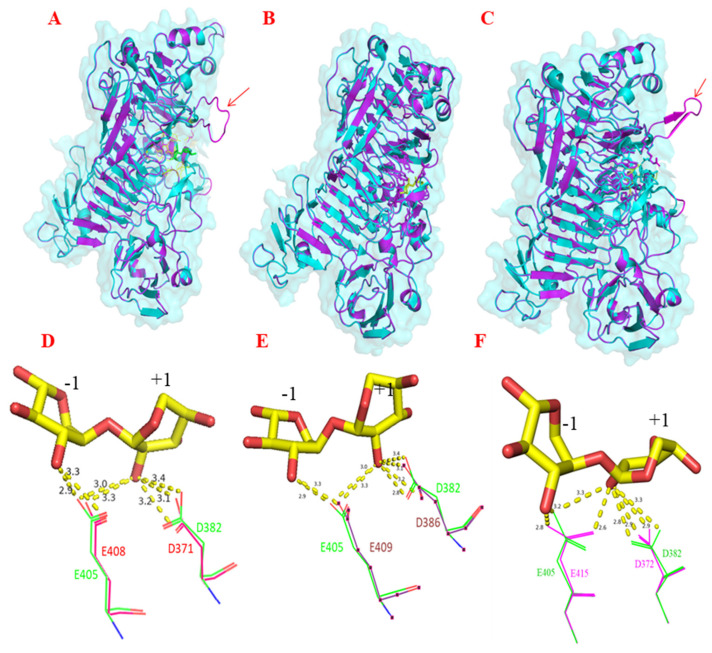
Homology modelling of Bp-XylX-1, Bp-XylX-2, and Lr-XylC β-xylosidases. Homology modelling of β-xylosidases from *L. reuteri* ATCC 53608 and *B. producta* ATCC 27340 was based on PDB ID: 3VSU of Ts-XOS using Phyre2. Bp-XylX-1 (**A**), Bp-XylX-2 (**B**), and Lr-XylC (**C**) are superimposed onto 3VSU (cyan). Catalytic nucleophile (Asp-D) and proton donor (Glu-E) were identified in Bp-XylX1 (**D**), Bp-XylX2 (**E**), and Lr-XylC (**F**) on the basis of 3VSU (green). Homology modelling was performed in PyMOL.

To gain more insights into the mechanism of action of these enzymes, structural models of Bp-XylX-1, Bp-XylX-2, and Lr-XylC were determined by homology modelling using a GH120 xylosidase (Ts-XOS) as a template. Bp-XylX-1, Bp-XylX-2, and Lr-XylC shared the same two-domain structure as present in Ts-XOS, with the major domain consisting of six right-handed *β*—helix repeats surrounded by five additional *β-*strands and seven α-helices (Figures S11–S13). An additional loop was observed in the Lr-XylC and Bp-XylX-1 domains (Figure 6). The minor domain was composed of a β-hairpin, three short α-helices, and two antiparallel β-sheets [50]. Amino acid residues forming hydrogen bonds within a 5 Å vicinity of the Ts-XOS ligand binding site (xylobiose) were identified in Bp-XylX-1, Bp-XylX-2, and Lr-XylC (Figures S11–S13). Based on this structural alignment, the amino acids corresponding to Asp382 (nucleophile) and Glu405 (general acid/base) of Ts-XOS were selected for site-directed mutagenesis and replaced by Ala in Bp-XylX-1, Bp-XylX-2, and Lr-XylC, leading to the loss of enzymatic activity of the corresponding recombinant mutants, Bp-XylX-1-D371, Bp-XylX-1-E408, Bp-XylX2-D386, Bp-XylX2-E409, Lr-XylC-D382, and Lr-XylC-E415, on *p*NP-Xyl (Table 1). Together with the product profiles of transxylosylation, these results suggested that Bp-XylX-1, Bp-XylX-2, and Lr-XylC cleaved the glycosidic bond in *p*NP-Xyl through a retaining mechanism (double-displacement mechanism) using Asp and Glu catalytic residues. In agreement with this mechanism of action, we showed using MEGA-x that Bp-XylX-1, Bp-XylX-2, and Lr-XylC clustered in a clade with characterised enzymes from the GH120 family (Figure S14).

### 2.3. Proteomics and In Silico Analyses Identified the Complement of Proteins Involved in XOS Utilisation across Lactobacilli and Blautia Strains from Different Hosts

In silico analysis of the *L. reuteri* ATCC 100-23C and *L. reuteri* ATCC 53608 genomes identified xylose utilisation genes adjacent to the xylosidase gene (*xylC*), including a xylulokinase (*xylG*), xylose isomerase (*xylF*), putative xylose repressor (ROK family protein, *xylR*), and glycoside-pentoside-hexuronide (GPH) family cation symporter/putative xyloside-cation symporter (*xylB*) (Table S3). An AraC-type DNA-binding domain (*xylA*) was also present in both *L. reuteri* ATCC 53608 and *L. reuteri* ATCC 100-23C genomes, which is likely to act as transcriptional regulator of xylanolytic gene expression, suggesting that in these *L. reuteri* strains, the genes were part of a cluster dedicated to XOS utilisation. In contrast, no XOS utilisation genes were identified adjacent to the xylosidases in the *B. producta* ATCC 27340 strain. A homologue of XylB in *B. producta* ATCC 27340 sharing 28% amino acid sequence similarity was observed (Table S3).

**Figure 7:**
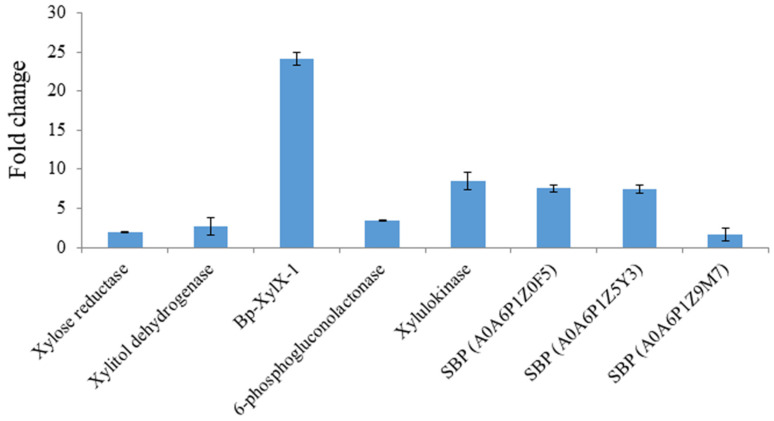
Proteomics analysis of *B. producta* ATCC 27340 grown on 1% XOS. Bacterium was grown on 1% XOS containing minimal medium, and proteomics analysis was performed on total intracellular proteins. Protein FASTA files for *B. producta* from UniProtKB (UP000464715; 5365 protein entries) was used for protein identification. SBP: solute-binding proteins of the ATP-binding cassette transporters (ABC transporters).

In order to further identify the proteins implicated in XOS utilisation in these strains, proteomics was carried on intracellular protein extracts from *L. reuteri* ATCC 53608, *L. reuteri* ATCC 100-23C, and *B. producta* ATCC 27340 grown on XOS. We observed many proteins of the predicted xylanolytic cluster in proteomes of *L. reuteri* ATCC 53608 and *L. reuteri* ATCC 100-23C, such as xylulokinase and xylose isomerase, a putative xylose repressor and GPH family cation symporter and major facilitator superfamily (MFS) transporter (Table S3). Additional proteins that could be involved in the degradation of plant polysaccharides were observed in the proteomes of *L. reuteri* ATCC 53608 and *L. reuteri* ATCC 100-23C, as listed in Table S3. All the observed proteins shared between 97 and 100% amino acid sequence similarity between *L. reuteri* ATCC 53608 and *L. reuteri* ATCC 100-23C (Table S3). Homologues of these proteins were also observed in *B. producta* ATCC 27340 (Table S3), including a xylose reductase (XR), xylitol dehydrogenase (XDH), and 6-phosphogluconolactonase (6-PGL) and solute-binding protein (Figure 7), while no protein corresponding to xylose isomerase could be identified in the proteome or genome of *B. producta* ATCC 27340. Three solute-binding proteins of the ATP-binding cassette transporters (ABC transporters) were overexpressed (2- to 8-fold) in proteome of *B. producta* ATCC 27340. Two of them (UniProt protein IDs: A0A6P1Z0F5 and A0A6P1Z5Y3), showing an 8-fold overexpression, are likely to be involved in XOS transport in *B. producta* ATCC 27340.

**Figure 8:**
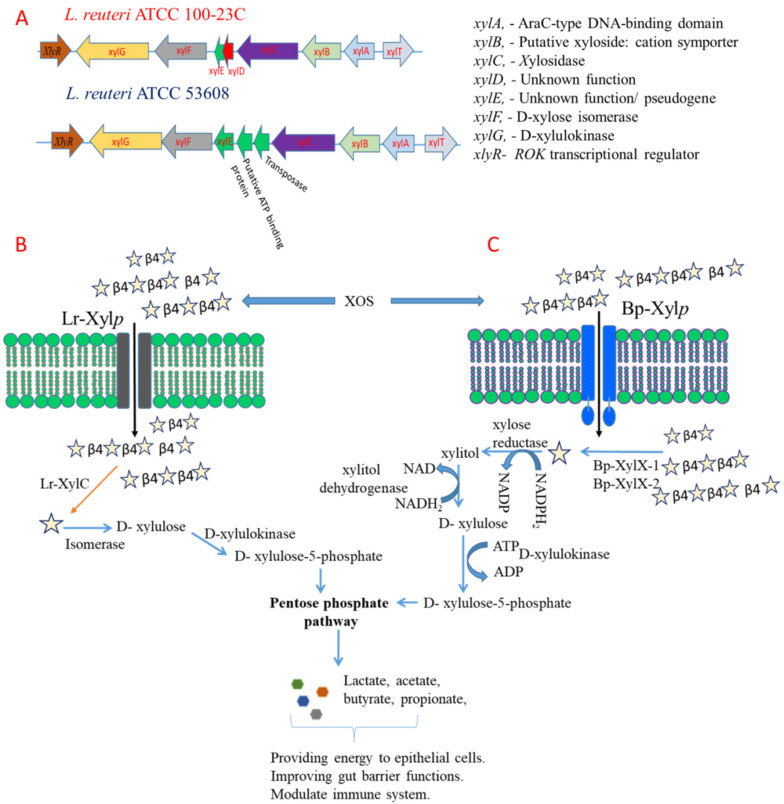
Identification of the XOS-utilising locus in L. reuteri and prediction of XOS metabolic pathways in L. reuteri and B. producta strains. Identification of the XOS-utilising locus in L. reuteri ATCC 53608 and ATCC 100-23C (**A**). Predicted metabolic pathway for XOS utilising in L. reuteri ATCC 53608 and ATCC 100-23C (**B**), and B. producta ATCC 27340 (**C**). During fermentation of XOS, these bacteria can produce SCFA, which can improve human health.

Based on these analyses, we proposed a model (Figure 8) in which XOS is taken up by *L. reuteri* ATCC 53608, *L. reuteri* ATCC 100-23C, and *B. producta* ATCC 27340 strains by a xyloside transporter (XylP), known in *Lactobacillus pentosus* to have a preference for xyloside-linked sugars rather than xylose [51]. XOS is then converted into xylose by cytoplasmic β-xylosidases Bp-XylX-1, Bp-XylX-2, and Lr-XylC. Then, in *L. reuteri*, a xylose isomerase (XylF) converts D-xylose into D-xylulose, which is further transformed to D-xylulose-5-phosphate by a xylulose kinase (XylG). In contrast, *B. producta* ATCC 27340 does not have an XylF, but an XR and XDH are predicted to convert xylose into D-xylulose. It is then likely that xylulose-5-phosphate enters the pentose phosphate pathway and produces short-chain fatty acids (SCFAs) as shown for *Lactococcus lactis* IO-1 [52]. The production of SCFAs from XOS fermentation has been previously reported in *Lactobacillus* [53,54]. Microbial fermentation of XOS has been shown to have a range of benefits for the host. For example, Hsu et al. [55] showed that dietary XOS supplementation in rats with precancerous colon lesions led to a modulation of the bacterial population and reduced number of aberrant crypt foci in the colon. In another study, mice fed with XOS and *Lactiplantibacillus plantarum* S2 showed a lower pH through production of acetate, which is beneficial to limit the growth of pathogenic bacteria such as *Enterococcus*, *Enterobacter*, and *Clostridia* species [56]. Moreover, SCFA produced through microbial fermentation of XOS has been shown to increase the strength of the intestinal barrier [57], reduce colonic [58] and adipose tissue inflammation [59], and ameliorate obesity induced by a high fat diet and glucose intolerance [60,61]. Such beneficial effects on gut health are therefore expected for *L. reuteri* and *B. producta* strains able to utilize XOS.

**Figure 9:**
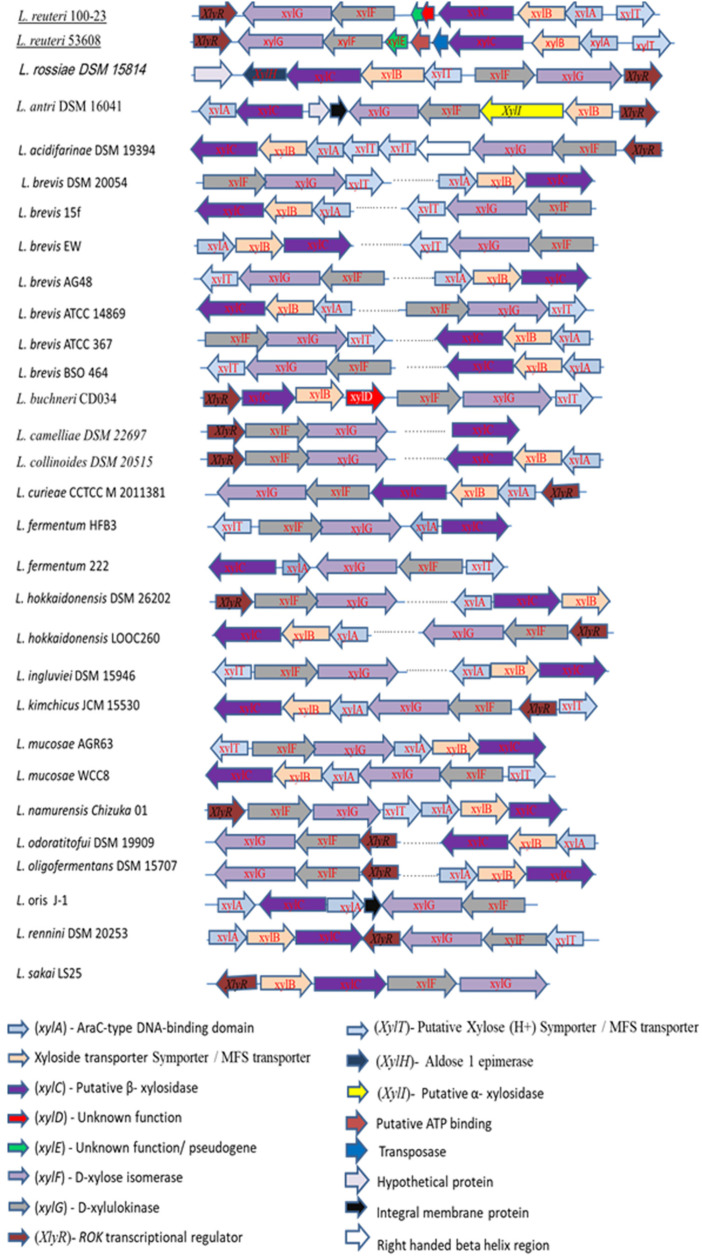
Synteny analysis of putative XOS-utilising gene organisation in *Lactobacilli* species.

Next, we screened for the presence of proteins encoded by the XOS utilisation cluster of *L. reuteri* ATCC 53608 and *L. reuteri* ATCC 100-23C using publicly available genomes of lactic acid bacteria at JGI/IMG, revealing 61 lactic acid bacteria containing at least the XylC gene out of the 796 genomes analysed. Homologous searches based on amino acid sequences of Lr-XylC indicated that xylosidases were highly conserved among lactobacilli isolated from different hosts. Lr-XylC shared the highest similarity with a putative xylosidase from *L. reuteri* strains (99% and 100% with *L. reuteri* mlc3 and *L. reuteri* ZLR003 isolated from rodent and piglet, respectively). Homologous searches extended to proteins present within the XOS locus of *L. reuteri* ATCC 53608 and *L. reuteri* ATCC 100-23C strains revealed differences in the nature, location, or orientation of the genes from this locus in different lactic acid bacteria (Figure 9). Screening of amino acid sequences of Bp-XylX-1 and Bp-XylX-2 against 169 *Blautia* species revealed 78 genomes of the *Blautia* harbouring xylosidase genes with an amino acid sequence similarity >50% to Bp-XylX-1 or Bp-XylX-2. Interestingly, Bp-XylX-1 shared 99% amino acid sequence similarity with homologous proteins in *B. producta* PMF1, *B. coccoides* DSM 935, and *B. coccoides* NCTC 11035. There was significant similarity among predicted xylosidases from *Blautia* species isolated from humans, chicken, and mice, suggesting that XOS utilisation is ubiquitous among *Blautia* species from different hosts. This further suggests that these host-associated *Lactobacillus* and *Blautia* strains acquired xylanolytic genes from a common ancestor during evolution.

In summary, we showed that *L. reuteri* ATCC 53608 and *B. producta* ATCC 27340 produced the β-D-xylosidases Lr-XylC, Bp-XylX-1, and Bp-XylX-2, enabling growth of these gut bacteria on XOS. These β-xylosidases shared conserved catalytic residues of the GH120 family, and displayed broad tolerance to pH, temperature, and xylose inhibition and the capacity to catalyse transxylosylation reactions as demonstrated by HPAEC, NMR, and ESI-MS. We also demonstrated that Bp-XylX-1 and Lr-XylC could degrade longer XOS efficiently, up to xylotriose and xylotetraose, than could Bp-XylX-2. In *L. reuteri* ATCC 53608 and ATCC 100-23C strains, these enzymes were part of a cluster genes involved in XOS utilisation, whereas the *B. producta* XOS metabolism pathway relied on enzymes/transporters spread in its genome, as also confirmed by proteomics. Synteny analysis suggested that the utilising potential of XOS is widely distributed among *Blautia* and *Lactobacillus* species, supporting the prebiotic potential of XOS in modulating the HGM in disease conditions.

## 3. Materials and Methods

### 3.1. Bacterial Strains, Media, Carbon Source, and Growth Assays

Commercially available xylooligosaccharides (XOS) from corncob were purchased from Carbosynth (Newbury, Berkshire—UK). Xylose, xylobiose, xylotriose, xylotetraose, arabinan, arabinoxylan, and xyloglucan were procured from Megazyme (Wicklow, Ireland). *L. reuteri* strain ATCC 20016, *L. reuteri* ATCC 100-23C, and *L. reuteri* ATCC 53608 isolated from the gut of humans, mice, and pigs, respectively, were from an in-house collection. *B. producta* ATCC 27340 (JCM 1471^T^) was purchased from the Japan Collection of Microorganisms (JCM-RIKEN BRC).

To determine the growth pattern of the bacteria, 5 µL of glycerol stocks of these strains were initially incubated in 20 mL of de Man, Rogosa, and Sharpe medium (MRS) or Gifu anaerobic medium (GAM) overnight at 37 °C. The next day, cells were harvested by centrifugation (3000× *g* at room temperature, RT) and washed thrice with 20 mL phosphate-buffered saline (PBS). The cells were resuspended in 20 mL PBS, and 5 µL of this suspension was used to inoculate 150 μL of modified MRS or minimal medium supplemented with XOS, arabinan, arabinoxylan, or xyloglucan as the sole carbon source (1%) on a multiwell plate, as described previously [62]. Bacterial strains were incubated at 37 °C for 30 (*L. reuteri* strains) or 56 h (*B. producta*) in an anaerobic growth chamber, and the O.D. was measured every hour or 8 h of intervals using a microplate reader at 595 nm.

### 3.2. Analysis of Xylooligosaccharides

XOS was analysed using matrix-assisted laser desorption/ionisation–time of flight (MALDI-TOF) mass spectrometry (MS), high-performance anion-exchange chromatography with pulsed amperometric detection (HPAEC-PAD), and nuclear magnetic resonance (NMR) spectroscopy. For MALDI-TOF-MS, acetylation was carried out in standard anhydrous pyridine and acetic anhydride (3:1) solvent with the addition of 4-dimethylaminopyridine as a catalyst. Following overnight incubation, the clear solution was treated with methanol and concentrated with toluene in vacuo. Flash chromatography was subsequently performed on silicycle 12 g columns (required precolumn to load sample) in toluene–acetone 0–30% (20 CV) + 30% (5 CV), affording moderate separation. Acetylated XOS were then fractionated by gel permeation chromatography (GPC, Toyopearl TSK-HW 40S resin in 100 cm × 2.2 cm column) and freeze-dried. A 5 mg quantity of the resulting dry powder was dissolved in 1 mL of Milli-Q water, and 5 µL thereof was mixed with an equal volume of 2, 5-dihydroxybenzoic acid matrix (10 mg/mL in 30% acetonitrile + 0.1% TFA in H_2_O). This mixture was spotted on a MALDI target plate (Bruker MTP 384 Polished Steel TF Target), and then analysed in positive acquisition mode on a Bruker Daltonics—Autoflex^™^ speed MALDI-TOF. All results were analysed using the Bruker FlexAnalysis software.

^1^H, ^13^C, DEPT, and HSQC-NMR spectra were recorded on a Bruker Avance III 400 MHz spectrometer equipped with a broadband BBFO probe. Chemical shifts (δ) were accounted for in parts per million (ppm) using the residual solvent signal for referencing. A Silica Gel 60 F254 (Merck) thin-layer chromatography (TLC) plate was used for checking the reaction of acetylation where the mobile phase was ethyl acetate, acetic acid, and water (V/V, 2:1:1).

The analysis of oligosaccharide profiles of pre- and post- fermented cell free supernatants from *L. reuteri* strains, collected at 0, 24, and 48 h, was carried out by HPAEC-PAD using a Dionex ICS-3000 system (Dionex, Sunnyvale, CA, USA). XOS (10 μL aliquots) were separated on a CarboPac PA100 (Dionex) analytical-exchange column with dimensions of 250 mm × 4 mm coupled with a CarboPac PA1 guard column (Dionex, dimensions 50 mm × 4 mm) on a pulsed electrochemical detector using PAD mode. The elution was performed at a constant flow rate of 1.0 mL min^−1^ at 30 °C using the following eluents for the analysis (A) 200 mM NaOH, (B) 100 mM NaOH and 550 mM NaOAc, and (C) Milli-Q water. The following linear gradients of sodium acetate were used: 0 mM; 0–51 min, 16 mM; 51–56 min, 100 mM; 56.1–61 min, 0 mM.

The analysis of oligosaccharide profiles of pre- and post fermented cell free supernatants from *B. producta* ATCC 27340, collected at 0, 24, 48, and 72 h, were carried out by high-performance anion-exchange chromatography with an electrochemical detector (HPAEC-ED) using a Dionex ICS-6000 DC (Thermo Fisher, Waltham, MA, USA). XOS (10 μL aliquots) were separated on a Dionex CarboPac^TM^ PA1 analytical-exchange column with dimensions of 250 mm × 4 mm, and signals were monitored in a pulsed electrochemical detector. The elution was performed at a constant flow rate of 1.0 mL min^−1^ at 30 °C using an isocratic flow of 200 or 150 mM NaOH. All HPAEC-PAD/ED results were analysed using Chromeleon (Thermo Fisher).

### 3.3. Proteomics

*L. reuteri* strain ATCC 53608 and *L. reuteri* strain ATCC 100-23C were grown in an anaerobic glove box, whereas *B. producta* ATCC 27340 was grown using an anoxomat anaerobic jar. Initial cultures of these strains were prepared by inoculating 5 µL of glycerol stocks in 10 mL of MRS for *L. reuteri* strains and GAM for *B. producta*. After overnight incubation at 37 °C, cells were collected by centrifugation (3000× *g* at RT) and washed thrice with 5 mL PBS. Cells were resuspended into 2 mL PBS, and 5 µL was used to inoculate 10 mL of modified MRS or minimal medium containing 2% XOS or glucose, respectively. The growth was monitored by measuring OD_595_ nm in a microtiter plate on a Tecan Infinite F50 microplate reader. After reaching the mid-late exponential phase (OD_595_ ~ 0.5–0.6) in two biological replicates, the cells were harvested by centrifugation (5000× *g*) for 5 min at 4 °C, washed twice with ice-cold PBS, and resuspended in 2 mL lysis buffer (50 mM HEPES containing protease inhibitors). For proteome analysis, cells were lysed by bead beating (60 s cycle for three times on bead beater homogenizer, MP Biomedicals, CA), using glass beads (acid washed ≤106 µm). Lysates were centrifuged (15,000× *g*, 15 min at 4 °C), the supernatant was collected, and protein concentrations were determined by a Bradford assay (Thermo Fisher Scientific). Peptides analyses from *L. reuteri* strains and *B. producta* ATCC 27340 were performed as per standard methods (details are provided in the Supplementary Materials).

### 3.4. In Silico Analyses

For phylogenetic analyses, amino acid sequences of β-xylanases and β-xylosidases belonging to GH families 10, 11, 43, and 120 were sourced via the CAZy database and/or from the National Center for Biotechnology Information (NCBI). These sequences were used to generate a phylogenetic tree based on the evolutionary history that was inferred by using the maximum-likelihood method and the Jones–Taylor–Thornton matrix-based model [63] in Molecular Evolutionary Genetics Analysis-X (MEGA-X) [64] software.

SignalP-5.0 Server [65] and PSORTb v3.0 [66] were used to predict the presence of signal peptides in proteins associated with XOS utilisation in the *L. reuteri* and *B. producta* strains. The presence or absence of an XOS locus was checked through the genome sequences of these strains publicly available in the Joint Genome Institute (JGI)/Integrated Microbial Genomes (IMG) system [67]. The presence of genes or loci for XOS utilisation was searched among the genomes of different *Lactobacillus* strains available in the JGI/IMG system for synteny analysis. The default threshold e-value was 1e-5, and a sequence comparison of genes or loci for XOS utilisation was conducted in which annotated protein sequences of each *L. reuteri* strain were subjected to BLASTP searches against other *Lactobacillus* members, and amino acid similarities <50% were excluded. Similar searches were performed for *Blautia* species using the JGI/IMG system server.

Amino acid sequences of *L. reuteri* and *B. producta* β-xylosidases were used to predict their tertiary structure using the Phyre2 Protein Fold Recognition Server [68]. Tertiary homology modelling of β-xylosidases and prediction of active site residues were determined by comparing a structure of β-xylosidase from the *Thermoanaerobacterium saccharolyticum* JW/SL-YS485, Ts-XOS (PDB ID: 3VSU), using the PyMOL software.

### 3.5. Cloning, Heterologous Expression, and Purification of Recombinant Enzymes

The *L. reuteri* ATCC 53608 and *B. producta* ATCC 27340 were grown overnight in MRS and GAM, respectively, following aforementioned procedures. The genomic DNA from *L. reuteri* ATCC 53608 was purified using the Monarch^®^ Genomic DNA Purification Kit (New England Biolabs) following the manufacturer’s instructions, with the following steps for cell lysis: 1 mL of culture was centrifuged at 10,000× *g,* and the cells were resuspended in 400 μL tissue lysis buffer. The suspension was then transferred into a lysing matrix B tube (MP Biomedicals), and cells were disrupted by 3 cycles of bead-beating at maximum speed (6.5 m/s, 50 sec) with 1 min rest between cycles. The genomic DNA from *B. producta* ATCC 27340 was isolated by using the GenElute™ bacterial genomic DNA Kit (NA2110-1KT, Sigma Aldrich, India) following the manufacturer’s instructions.

The purity of the genomic DNA was checked on 0.8% agarose gels, and the concentration determined with a NanoDrop spectrophotometer (Thermo Fisher Scientific). Primers were designed excluding lapidated [69] and signal peptide amino acid regions from the gene if present. Restriction sites were carefully optimised after ensuring that there were no restriction sites within the gene for selected restriction enzymes (Table S1). Primers were synthesised by Eurofins Scientific (India) and used in PCR reactions (50 μL) containing 25 μL of Master Mix with Q5^®^ High-Fidelity DNA Polymerase, 10 ng of each primer, and 50 ng of purified DNA. The PCR included an initial denaturation step at 98 °C for 5 min, followed by 30 cycles at 98 °C for 45 s, 65/66/64 °C (annealing temperature for different primers) for 30 s, and 72 °C for 3 min, with a final 5 min extension at 72 °C. Following purification, the PCR products and expression vector pET28a (Novagen) were digested with NheI/XhoI or NdeI/XhoI and used in ligation reactions. The constructs were transformed into *Escherichia coli* TOP10 cells, and positive colonies were selected by colony PCR. The plasmids were then isolated using a GeneJET Plasmid Miniprep Kit (Thermo Fisher cat. no.: K0503), and the integrity of the inserts determined by restriction digestion and Sanger sequencing using 3730xl DNA Analyzer and the same primers. The BigDye^®^ Terminator v3.1 Cycle Sequencing Kit (Applied Biosystems) was used for sequencing. After confirmation, the recombinant plasmids were transformed into Rosetta™ competent cells (*E. coli* BL21 (DE3) for protein expression.

The recombinant Rosetta cells were cultured at 37 °C in Luria–Bertani (LB) medium with chloramphenicol (35 μg/mL) and kanamycin (50 μg/mL) until the OD_600_ reached 0.6. The expression of the recombinant proteins was induced by adding a final concentration of 0.5 mM isopropyl β-D-1 thiogalactopyranoside (IPTG). The culture was further incubated at 18 °C for 16 h, and bacterial cells were then harvested by centrifugation at 5000× *g* for 10 min at 4 °C. The bacterial pellet was resuspended in phosphate buffer (50 mM, 300 mM NaCl, pH 7.4) with an appropriate cOmplete™, EDTA-free Protease Inhibitor Cocktail (Sigma Aldrich, cat. no. 4693159001). Cells were lysed by ultrasonication using a cycle of 10 min (3 s on and 5 s off) on ice, and the supernatant was collected following centrifugation at 16,000× *g* for 30 min at 4 °C. The supernatant was then concentrated to 2–3 mL using an Amicon^®^ Ultra-15 Centrifugal Filter Unit (10 kDa), mixed with 50 mM sodium phosphate buffer (pH 7.4—buffer A) containing 300 mM NaCl, and incubated for 30 min at 4 °C with cOmplete™ His-Tag Purification Resin. The mix was then poured into a column (20 mL in length) and washed with 50 mL buffer A. The His-tagged bounded protein was eluted with a 50 mM sodium phosphate buffer (pH 7.4; buffer B) containing 150 mM imidazole and 300 mM NaCl. The fractions containing the recombinant protein were pooled, and the imidazole was removed by centrifugation with an Amicon^®^ Ultra-15 Centrifugal Filter Unit (10 kDa) using 10 mM sodium phosphate buffer (pH 5.7). Finally, the concentration of the recombinant protein was determined using a Bradford assay. The molecular weight and purity of the recombinant proteins was determined by sodium dodecyl sulfate polyacrylamide gel electrophoresis using 10% polyacrylamide gels, and the proteins were stored at −80 °C until use.

### 3.6. Enzymatic Activity Assays

Cell lysates and supernatants of *L. reuteri* ATCC 100-23C, *L. reuteri* ATCC 53608, and *B. producta* ATCC 27340 were prepared for checking enzymatic activity against a variety of artificial substrates. In brief, following overnight growth of bacteria under anaerobic conditions in a minimal medium containing 2% XOS, the culture supernatant was collected by centrifugation at 10,000× *g* for 30 min, and filtered with a 0.22 µm syringe filter. The bacterial pellets were resuspended in 1 mL of 50 mM potassium buffer (pH 6.5, containing 2 mM DTT and cOmplete™, EDTA-free Protease Inhibitor Cocktail) and then transferred into 2 mL of screw-cap tubes containing 0.3 g glass beads (≤106 µm, Sigma Aldrich, UK). The bacterial cells were then disrupted using a bead beater at maximum speed for three cycles of 1 min speed and 1 min interval incubation on ice. Following centrifugation at 15,000× *g* for 30 min, the cell lysates were collected and stored on ice. Proteins were concentrated using a 3000 molecular weight cut-off (Amicon^®^ Ultra-15 Centrifugal Filter Unit) and immediately used for enzymatic activity assay.

The 1-4 β-xylosidase and 1-4 β-xylanase activity assays were carried out using *p*-nitrophenyl (*p*NP) β-D-xylopyranoside (Xyl) (Megazyme) and 4,6-*O*-(3-ketobutylidene)-4-nitrophenyl-β-D-4^5^-glucosyl-xylopentaoside (K-Xyl6-*p*NP) (Megazyme) substrates, respectively. The reactions were performed in 50 µL of 50 mM sodium phosphate buffer (pH 6.5) with 2 mM of *p*NP-Xyl or K-Xyl6-pNP and 0.3 mg/mL (cell lysate or supernatant) at 37 °C for 10 min with *p*NP-Xyl and 30 min with K-Xyl6-pNP. Different artificial substrates such as *p*NP-β-D-glucopyranoside (*p*NP-Glc), *p*NP-β-D-cellobioside (*p*NP-Cello), *p*NP-N-acetyl-β-D-glucosaminide (*p*NP-GlcNAc), *p*NP-β-D-galactopyranoside (*p*NP-Gal), *p*NP-β-D-mannopyranoside (*p*NP-Mann), and *p*NP-β-D-laminaribiose (*p*NP-laminaribiose) were also used (at 2 mM concentration) to assess the broad spectrum activity of the cell lysates and supernatants and recombinant enzymes. The reactions were terminated by adding 6 volumes of 1 M Na_2_CO_3_, and the released *p*-nitrophenol was determined by measuring the absorbance at 410 nm on a SpectraMax^®^ i3x Multi-Mode Microplate Reader. The concentration of released *p*-nitrophenol was determined accordingly to the Beer–Lambert law (A = ε *l* c, where ε is the molar extinction coefficient of *p*NP at 410 nm, *l* is the optical path length in cm, and c- is the concentration). The extinction coefficient for *p*NP was determined against a standard curve of different concentrations of *p*NP in a 50 mM sodium phosphate buffer (pH 6.5) at 37 °C, and was found to be 1000 M^−1^ cm^−1^.

To determine the optimum pH, the recombinant enzymes (1.3 µM) were incubated at 37 °C for 10 min with 2 mM *p*NP-Xyl as a substrate in 100 µL reactions using the following buffers: citrate (pH 3 to 5.5, 50 mM), sodium phosphate (pH 5.7 to 8, 50 mM), and Tris-HCl (pH 9, 50 mM). To determine the optimum temperature, the enzymatic reactions were carried out at different temperatures (5, 10, 15, 20, 25, 30, 37, 48, 55, 60, 65, 70, and 75 °C) in sodium phosphate buffer (50 mM, pH 5.7) with 2 mM *p*NP-Xyl and 1.3 µM enzymes. The Michaelis–Menten kinetics parameters of the recombinant enzymes were determined using various concentrations of *p*NP-Xyl. Reactions (100 µL) containing *p*NP-Xyl and 1.3 µM enzymes in 10 mM sodium phosphate buffer (pH 5.7) were incubated at 45 °C for 10 min. The reactions were terminated, and the released quantity of *p*-nitrophenol was determined as described above.

The enzymatic activity of the recombinant enzymes against XOS (xylobiose, xylotriose, and xylotetraose) was performed in 100 µL sodium phosphate buffer (50 mM, pH 5.7) containing 10 mM substrate and 1.3 µM enzyme at 45 °C for various times (15 min, 30 min, 1 h, 2 h, 4 h, 8 h, and overnight). After each incubation time, the reaction was terminated by heating to 100 °C for 5 min and then analysed by TLC and HPAEC-ED. TLC was conducted using butanol:ethanol:water (5:3:2, *v*/*v*/*v*) as a mobile phase, and sugars were visualised by spraying the TLC with 5% H_2_SO_4_ in ethanol, followed by heating. For HPAEC-ED, a Dionex CarboPac PA1 guard column (2 mm × 50 mm) and Dionex CarboPac PA1 analytical column (4 mm × 250 mm) were used with a flow rate of 1 mL/min, using 200 or 150 mM NaOH as a mobile phase in an isocratic mode.

Transglycosylation activity assays were performed by adding 10 µL methanol or ethanol in the reaction containing 2 mM *p*NP-Xyl and 10 µL of enzyme (1.3 µM) in a total volume of 100 reaction of 50 mM sodium phosphate buffer (pH 5.7). Reactions were incubated for 1 h at 45 °C. After incubation, the reaction mixture was concentrated and run on TLC as described above. Transglycosylation activity was also confirmed by electrospray ionisation mass spectrometry (ESI-MS) using A 5500 QTRAP mass spectrometer (AB Sciex, Foster City, CA, USA) with a method established previously [62].

The susceptibility of the recombinant enzymes to inhibition by xylose was determined in the presence of different concentrations (0 to 400 mM) of xylose in the standard reaction mixture (100 µL), containing 2 mM *p*NP-Xyl and 10 µL of an enzyme (1.3 µM) in 50 mM sodium phosphate buffer (pH 5.7). The reaction mixture was incubated at 45 °C for 5 min, and termination and reading of all reactions were carried out as described above.

### 3.7. Fluorophore-Assisted Carbohydrate Electrophoresis (FACE)

Xylobiose, xylotriose, and xylotetraose were incubated with recombinant β-xylosidases for 15 min in 10 mM sodium phosphate buffer (pH 5.7) at 45 °C. The enzymatic reactions were terminated by heating at 100 °C for 5 min, followed by centrifugation at 15,000× *g* for 10 min. The supernatant (10 µL) was then mixed with 10 µL of 0.2 M 7-amino-1,3-naphthalenesulfonic acid (Sigma Aldrich, India) prepared in 15% acetic acid. Following incubation at RT for 1 h in the dark, 10 µL of 1 M sodium cyanoborohydride (prepared in dimethyl sulfoxide, Sigma Aldrich) was added to the mixture, and further incubation was carried out overnight at 37 °C. The samples were then freeze-dried and resuspended in 20% glycerol. Samples (2.5 µL) were run in a 30% native polyacrylamide gel at 15 mA for around 3 h, and bands were visualised using a UV transilluminator.

### 3.8. Site-Directed Mutagenesis

Site-directed mutagenesis was carried out using the Q5^®^ Site-Directed Mutagenesis Kit (cat. no. E0554S). The wild-type plasmid Bp-XylX-1-pET28a (+), Bp-XylX-2-pET28a (+), and Lr-XylC-pET28a (+) were used as template, and the forward and reverse primers were designed using NEBaseChanger™ with slight modifications (Table S2). The PCR 10 µL mix contained in the final concentration of Q5^®^ Hot Start High-Fidelity 2X Master Mix was 1X, 0.5 µM of each primer, and 14 ng DNA, and the rest of the volume was made up by nuclease-free water. PCR was performed according to the following steps: initial denaturation at 98 °C for 1 min, and each cycle run consisted of 10 s of denaturation, 20 s of annealing, and a 7 min extension at 72 °C. The final extension was 5 min, and 25 cycles were used in this experiment. The PCR products were verified on 0.8% agarose gels and then used in ligation reactions. KLD (enzyme mix of kinase, ligase, and DpnI) (5 µL) was added to the reaction mixture and incubated for 1 h at RT to cleave the methylated template DNA. Following transformation of *E. coli* TOP-10 cells, the plasmids were isolated and sequenced as described above. The recombinant plasmids harboring the mutation were transformed into Rosetta™ (DE3) competent cells for protein expression. The recombinant enzymes were expressed and purified as described above.

### 3.9. Statistical Analyses

Enzymatic kinetic parameters (Km, k_cat_, and catalytic efficiency) were determined using GraphPad Prism, version 5. Two-way ANOVA and Bonferroni post-tests were also performed using GraphPad Prism, version 5. The standard error for enzymatic assays was determined using Microsoft Excel.

## Figures and Tables

**Table 1:** Kinetic analysis of recombinant β-D-xylosidases from *L. reuteri* and *B. producta* strains using pNP-Xyl as substrate, determined in 50 mM sodium phosphate buffer (pH 5.7) at 45 °C.

Enzyme	K_m_ (mM)	k_cat_ (s^−1^)	k_cat_/K_m_ (s^−1^mM^−1^)
Bp- XylX-1	0.19 ± 0.02	11.9 ± 0.23	63
Bp-XylX-2	0.19 ± 0.02	10.7 ± 0.2	56
Lr-XylC	0.23 ± 0.02	11.4 ± 0.2	49
Bp-XylX-1-D371	0	0	0
Bp-XylX-1-E408	0	0	0
Bp-XylX-2-D386	0	0	0
Bp-XylX-2-E409	0	0	0
LR-XylC -D382	0	0	0
LR-XylC -E415	0	0	0

## Data Availability

Not applicable.

## Supplementary Materials

The following supporting information can be downloaded at: https://www.mdpi.com/article/10.3390/ijms23062992/s1.
