# Supplementary material for: Biochemical Basis of Xylooligosaccharide Utilisation by Gut Bacteria
Source: Int J Mol Sci. 2022 Mar 10;23(6):2992. doi: 10.3390/ijms23062992 (PMC8954004; doi:10.3390/ijms23062992)
Supplement: Supplementary file 1 [file ijms-23-02992-s001.zip › ijms-1571188-supplementary.pdf]

Biochemical basis of xylooligosaccharide utilisation by gut bacteria

Ravindra Pal Singh[1,2]*, Raja Bhaiyya[1], Raksha Thakur[1], Jayashree Niharika[1], Chandrajeet Singh[1,†], Dimitrios Latousakis[3], Gerhard Saalbach[2], Sergey A. Nepogodiev[2], Parveen Singh[4], Sukesh Chander Sharma[5], Shantanu Sengupta[4], Nathalie Juge[3] and Robert A Field[2,*‡]

1. Division of Food and Nutritional Biotechnology, National Agri-Food Biotechnology Institute (NABI), SAS Nagar, Punjab, 140306, India
2. Department of Biological Chemistry, John Innes Centre, Norwich Research Park, Norwich, NR47UH, UK
3. The Gut Microbes and Health Institute Strategic Programme, Quadram Institute Bioscience, Norwich Research Park, Norwich NR4 7UQ, UK
4. CSIR-Institute of Genomics and Integrative Biology, Mathura Road, New Delhi, 110025, India
5. Department of Biochemistry, Panjab University South Campus, Chandigarh, 160014, India

[†]Present address: Department of Biochemistry and Molecular Genetics, University of Virginia, Charlottesville, VA 22908 USA

[$]Present address: Department of Chemistry and Manchester Institute of Biotechnology, The University of Manchester, 131 Princess Street, Manchester M1 7DN, UK

*For corresponding authors:

Dr. Ravindra Pal Singh: r.p.singh@nabi.res.in

Prof. Robert A Field: robert.field@manchester.ac.uk

1. Proteomics of *L. reuteri* ATCC 100-23C, *L. reuteri* ATCC 53608 and *B. producta* ATCC 27340

1.1. Proteomics for *L. reuteri* strains

Total intracellular proteins from *L. reuteri* strains were precipitated from 200 µl of the above-prepared supernatant using methanol/chloroform (Wessel & Flugge, 1984). The pellets were dissolved in 140 µl of 20 mM sodium phosphate buffer (pH8) containing 1% sodium deoxycholate (SDC). Samples of ~50 µg proteins were mixed with 4 µl of 25 mM dithiothreitol (DTT) and incubated at 60 °C for 30 min. Alkylation of protein was carried out by adding 3 µl of 50 mM iodoacetamide (IAA) and then incubated for 15 min in the dark at RT. Trypsin digestion of protein was achieved by adding 5 µg trypsin (MS grade, Promega) to the above mixture and incubation at 37 °C for 16-18 h. Next, samples were diluted 1:1 with 2% (w/v) trifluoroacetic acid (TFA) to quench enzymatic activity and peptides were processed for mass spectrometry analysis. The SDC was removed by acid precipitation with 5% formic acid.

The *L. reuteri* peptides were analysed by nano LC-MS/MS on an Orbitrap Fusion™ Tribrid™ mass spectrometer coupled to an UltiMate® 3000 RSLCnano LC system (Thermo Fisher Scientific, Hemel Hempstead, UK). Briefly, the samples were loaded and trapped using a pre-column with 0.1% TFA at 20 µl min^{-1} for 3 min. The trap column was then switched in-line with the analytical column (nanoEase M/Z column, HSS C18 T3, 100 Å, 1.8 µm; Waters, Wilmslow, UK) for separation, using the following gradient of solvents A (water, 0.05% formic acid) and B (80% acetonitrile, 0.05% formic acid) at a flow rate of 0.3 µl min^{-1}: 0-3 min 3% B (trap only); 3-4 min linear increase B to 7%; 4-103 min increase B to 50%; 103-108 min increase B to 65%; followed by a ramp to 99% B and re-equilibration to 3% B. Data acquisition condition on an Orbitrap Fusion™ Tribrid™ mass spectrometer in positive ion mode: MS1/OT: resolution 120 K, profile mode, mass range *m/z* 300-1800, AGC 4e^5, fill time 50 ms; MS2/IT: data dependent analysis was performed using parallel CID/HCD fragmentation with the following parameters: top 20 in IT rapid, centroid mode, isolation window 1.6 Da, charge states 2-7, threshold 2.5e^4, CE = 30, AGC target 1.0e^4, maximum inject time was 35 m, dynamic exclusion 2 counts, 30 s exclusion, exclusion mass window ±7 ppm.

Recalibrated peak lists were generated with MaxQuant 1.5.8.3 (Tyanova, *et al.*, 2016) in label-free quantitation (LFQ) mode using the *L. reuteri* protein sequence database (UniProt, 20170418, 2191 entries) plus the MaxQuant contaminants database (245 entries). The quantitative LFQ results from MaxQuant with default parameters were used together with search results from an in-house Mascot Server 2.4.1 (Matrix Science, London, UK) on the same databases. For this search, a precursor tolerance of 6 ppm and a fragment tolerance of 0.6 Da were used. The enzyme was set to trypsin/P with a maximum of 2 allowed missed cleavages; oxidation (M), acetylation (protein N-term) and deamidation (N, Q) were set as variable modifications and carbamido-methylation (C) as fixed modification. The Mascot search results were imported into Scaffold 4 (www.proteomsoftware.com) using identification probabilities of 99% for proteins and 95% for peptides.

1.2. Proteomics for *B. producta*

For total intracellular protein from *B. producta* ATCC 27340, the supernatant was precipitated by adding cold trichloroacetic acid (final concentration of 20%) and then incubated for 1 h on ice. Afterwards, the precipitate was collected by centrifugation (15,000 × g, 15 min at 4 °C). The pellet of proteins was washed three times with 500 µl ice-cold 0.01 M HCl in 90% acetone and a residual amount of acetone was evaporated by incubating at RT. Precipitated proteins were suspended in 0.1 M Tris-HCl containing 8 M urea at pH 8.5. Reduction, alkylation and trypsin digestion were performed as described above for *L. reuteri* strains.

B. producta ATCC 27340 peptides were first cleaned-up using Oasis HLB 1 cc Vac cartridges (Waters) using the manufacturer's protocol. SWATH-MS analysis of each sample was performed on a quadrupole-TOF hybrid mass spectrometer (TripleTOF 6600, SCIEX) coupled to an Eksigent Nano LC-425 system. A SWATH-MS method was created with 93 precursor isolation windows, defined based on precursor m/z frequencies in DDA run using the SWATH Variable Window Calculator (SCIEX), with a minimum window of 5 m/z. Analysis parameters were optimised before any sample was run. Curtain gas and nebulizer gas were maintained at 25 and 20 psi respectively. The ion spray voltage and temperature was set to 5.5 kV and 250 °C respectively. About 4 µg peptides were loaded on a trap-column (ChromXP C18 CL 5 µm 120 Å, Eksigent, SCIEX) and desalting was performed with a flow rate of 10 µl per min for 10 min. Peptides were separated on a reverse-phase C18 analytical column (ChromXP C18, 3 µm 120 Å, Eksigent, SCIEX) in a 57 min long buffer gradient with a flow rate of 5 µl/min using water with 0.1% formic acid (buffer A) and acetonitrile with 0.1% formic acid (buffer B) as follows: 0-38 min 3% B; 3-38 min linear increase B to 25%; 38-43 min increase B to 32%; 43-45 min increase B to 80%; followed by a ramp to 90% B and re-equilibration to 3% B.

Samples were analysed in data-independent acquisition (DIA) mode, which was acquired using Analyst TF 1.7.1 Software (SCIEX). Accumulation time was set to 0.25 s for the MS scan (400–1250 m/z) and 0.025 s for the MS/MS scans (100–1500 m/z). Rolling collision energies were applied for each window based on the m/z range of each SWATH and a charge 2+ ion with a collision energy (CE) spread of 5. The total cycle time was 2.3 s. The SWATH-MS run files were analysed in Spectronaut^{TM} 15.4 software (Biognosys) using directDIA^{TM} library-free workflow. The Pulsar search engine was used for protein identification, enzyme cleavage rules were set to trypsin, carbamidomethylation was set as the fixed modification and protein N-terminal acetylation and oxidation (M) were set as variable modifications. Protein FASTA files for *B. producta* from UniProtKB (UP000464715, 5365 protein entries) was used and protein identification was performed with 0.05 FDR, while a mutated decoy method was used for FDR analysis. Direct DIA^{TM} analysis was performed with default settings and quantitation was performed using area at MS2 level. Cross-run data normalisation was performed where the normalisation strategy was set to automatic. Quantitative data were exported in the form of 'Run Pivot Report' and differential protein analysis was performed in Microsoft Excel.

A

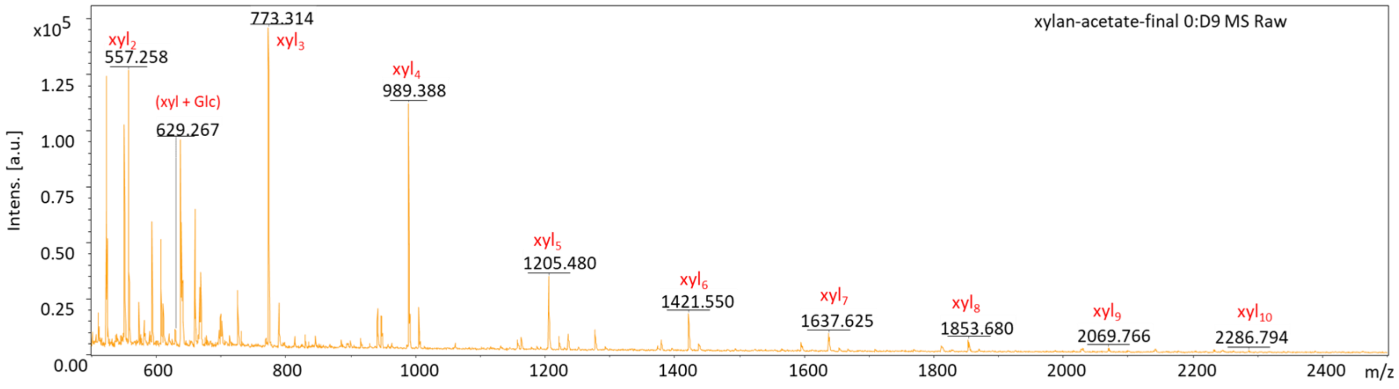

B

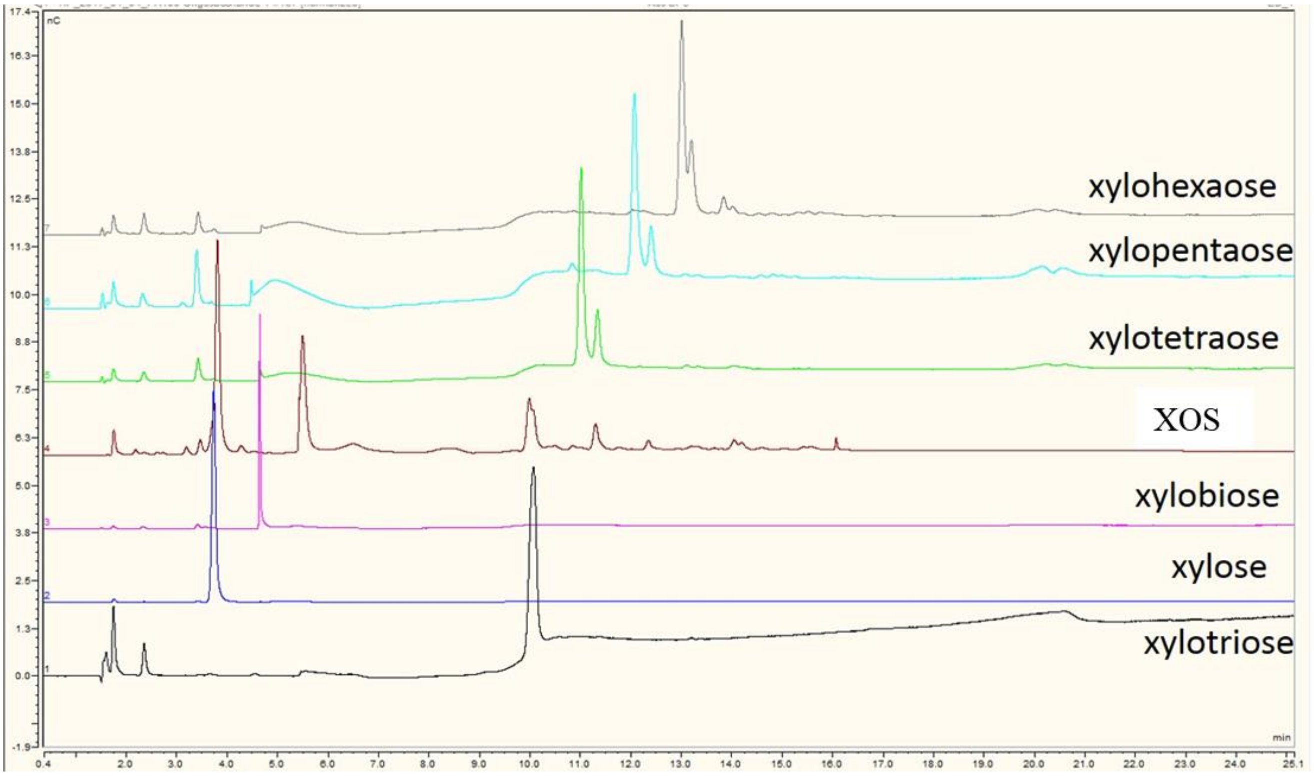

Figure S1: Chemical characterisation of XOS used in this study. MALDI-MS (A) and HPAEC-PAD (B).

A

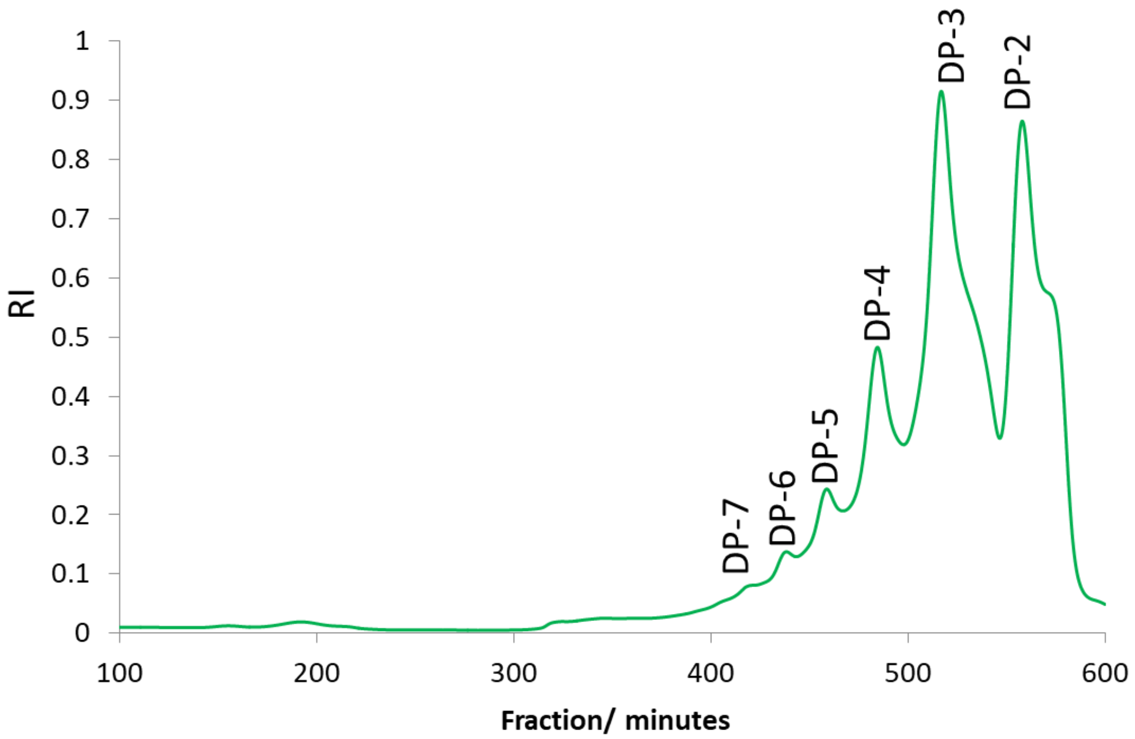
RI
Fraction/ minutes
DP-7
DP-6
DP-5
DP-4
DP-3
DP-2
0
0.1
0.2
0.3
0.4
0.5
0.6
0.7
0.8
0.9
1
100
200
300
400
500
600

B

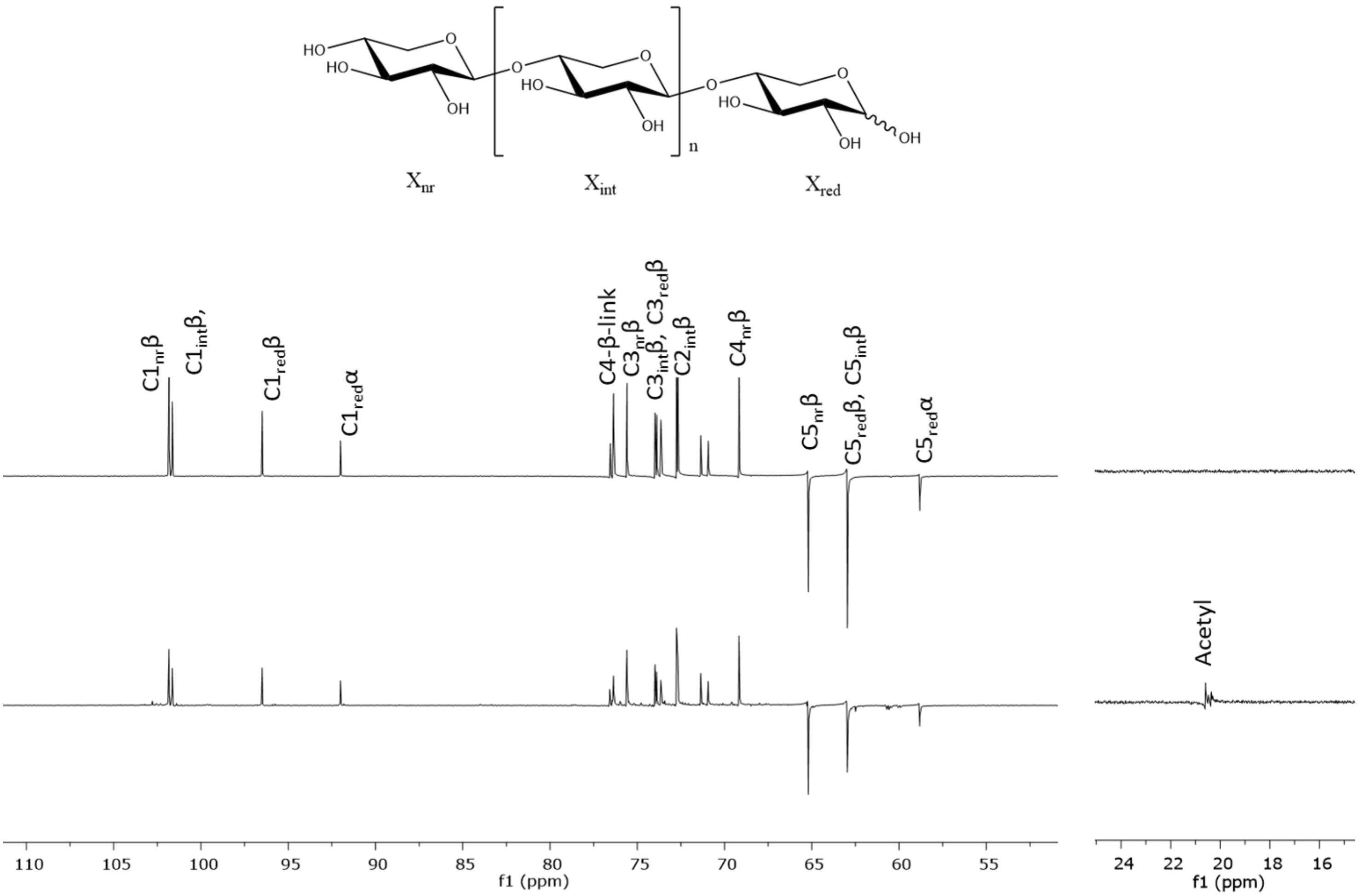
X_nr
X_int
X_red
n
C1_nrβ
C1_intβ,
C1_redβ
C1_redα
C4-β-link
C3_nrβ
C3_intβ, C3_redβ
C2_intβ
C4_nrβ
C5_nrβ
C5_redβ, C5_intβ
C5_redα
Acetyl
f1 (ppm)
110
105
100
95
90
85
80
75
70
65
60
55
24
22
20
18
16

C

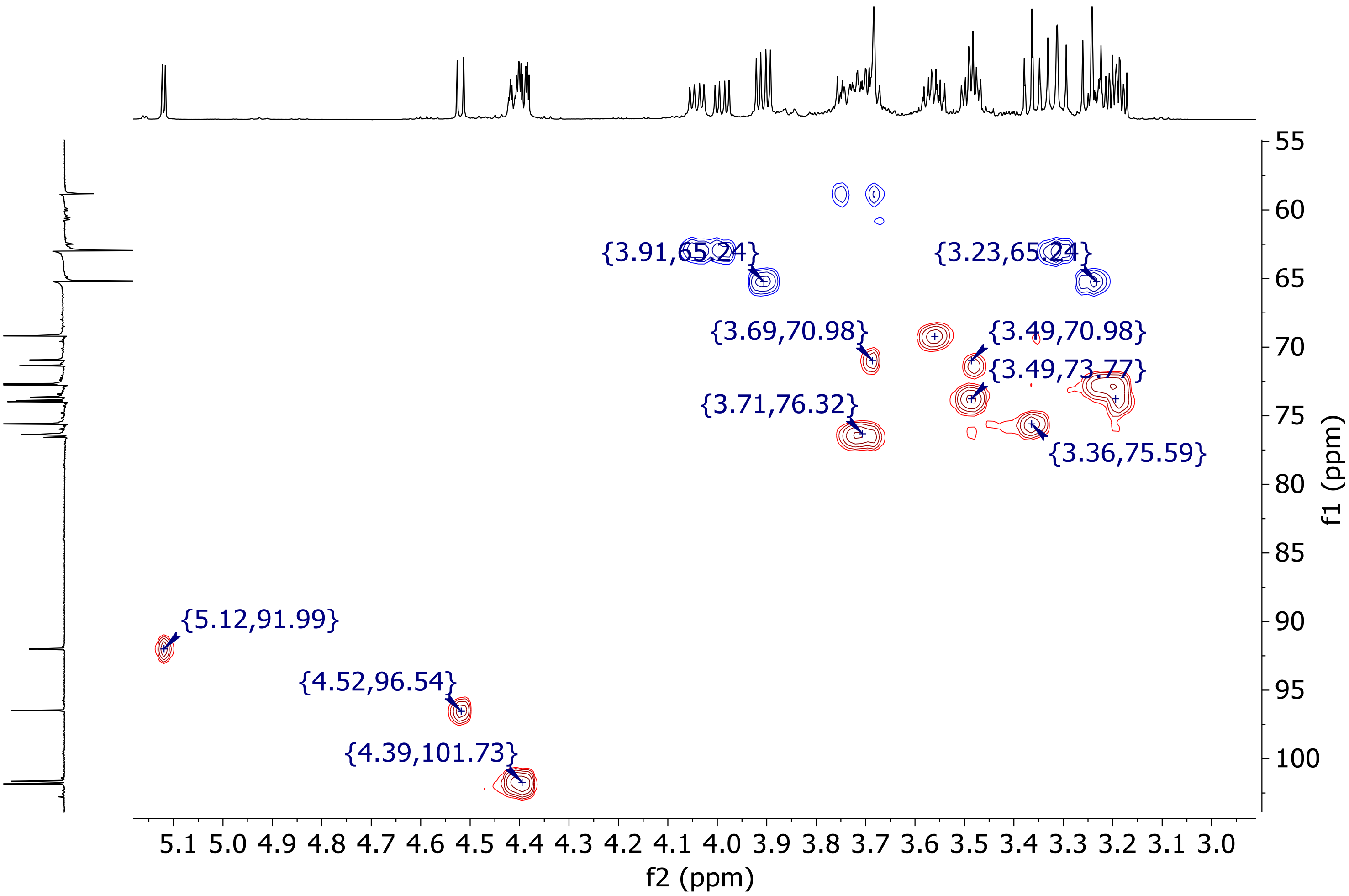

Figure S2: Chemical characterisation of XOS used in this study. (A) Gel permeation chromatography, (B) DEPT135-NMR of with and without acetate derivatives of XOS and (C) HSQC spectrum of XOS. DEPT135 of XOS with and without acetate derivatives clearly indicate that used XOS for bacterial growth was not substituted with acetyl group whose signal is present around 20.5 ppm. HSQC and DEPT135 identified reducing (red), non-reducing (nr) and internal (int) residues of XOS with all main signals located between 58.81 to 101.83 ppm. Anomeric region of XOS can be assigned at 101.83 ($C1_{nr}\beta$), 101.64 ($C1_{int}\beta$), 96.49 ($C1_{red}\beta$) and 92.00 ($C1_{red}\alpha$). The presence of signals for $C1_{red}\beta$ and$C1_{red}\alpha$ highlighted that the reducing end residue was unsubstituted and could freely be rotated in α and β conformations. The signals corresponding to (β-1,4) xylosidic linked residues were observed at 76.42 ($C4_{red}\beta$) and 76.36 ($C4_{int}\beta$) whilst signal for $C4_{red}\beta$ of non-reducing end residue was observed at 69.17 ppm. This analysis was in alignment with previously characterised XOS (Xiao, *et al.*, 2018).

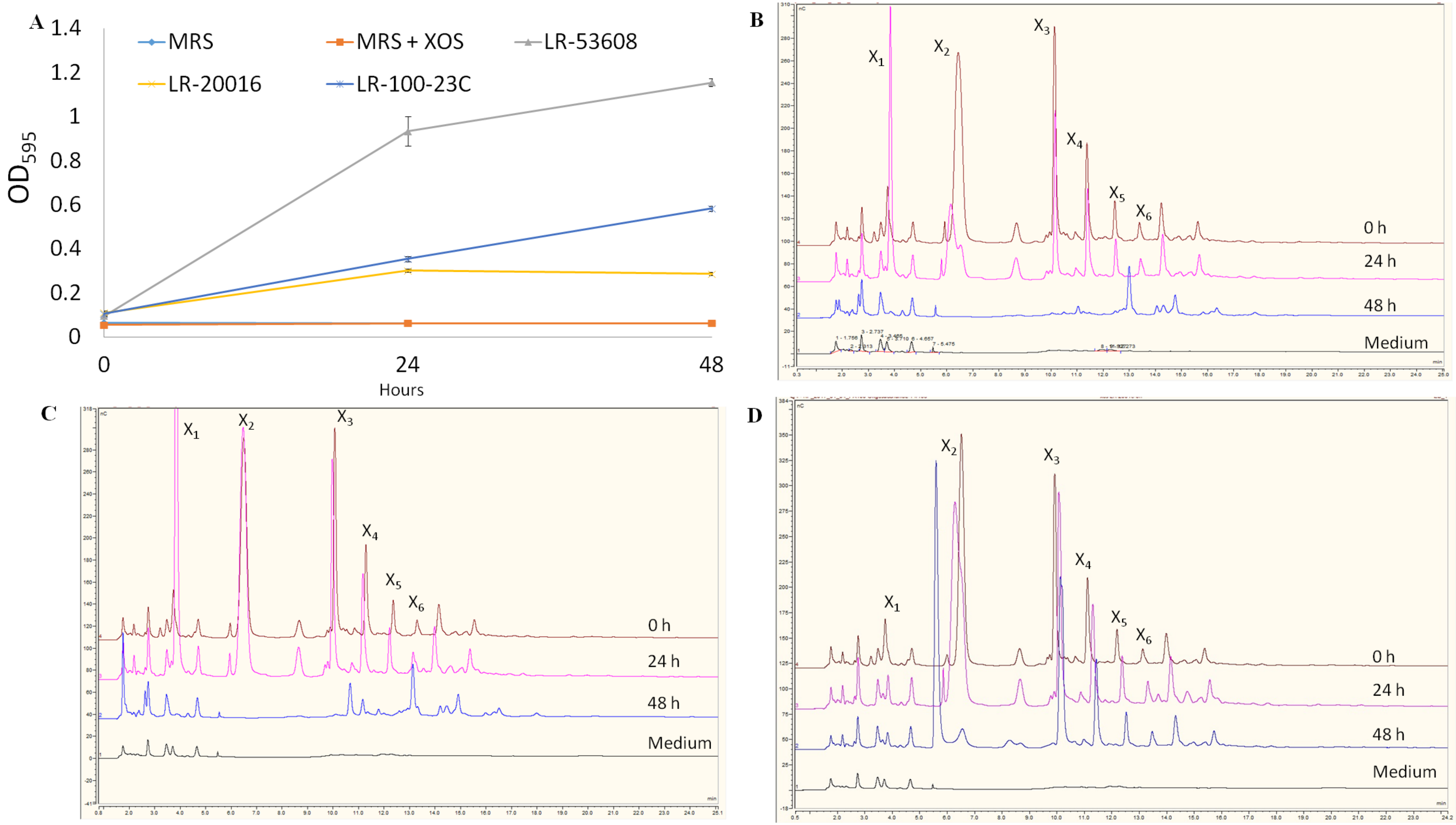

Figure S3: Analysis of *L. reuteri* XOS growth and utilisation. (A) Growth curves of *L. reuteri* strains cultured in modified MRS containing 1% XOS (MRS+ XOS). HPAEC analysis of XOS utilisation by *L. reuteri* ATCC 53608 (B), *L. reuteri* ATCC 100-23C (C) and *L. reuteri* ATCC 20016 (D). X_1- xylose, X_2- xylobiose, X_3- xylotriose, X_4- xylotetraose, X_5- xylopentaose and X_6- xylohexaose.

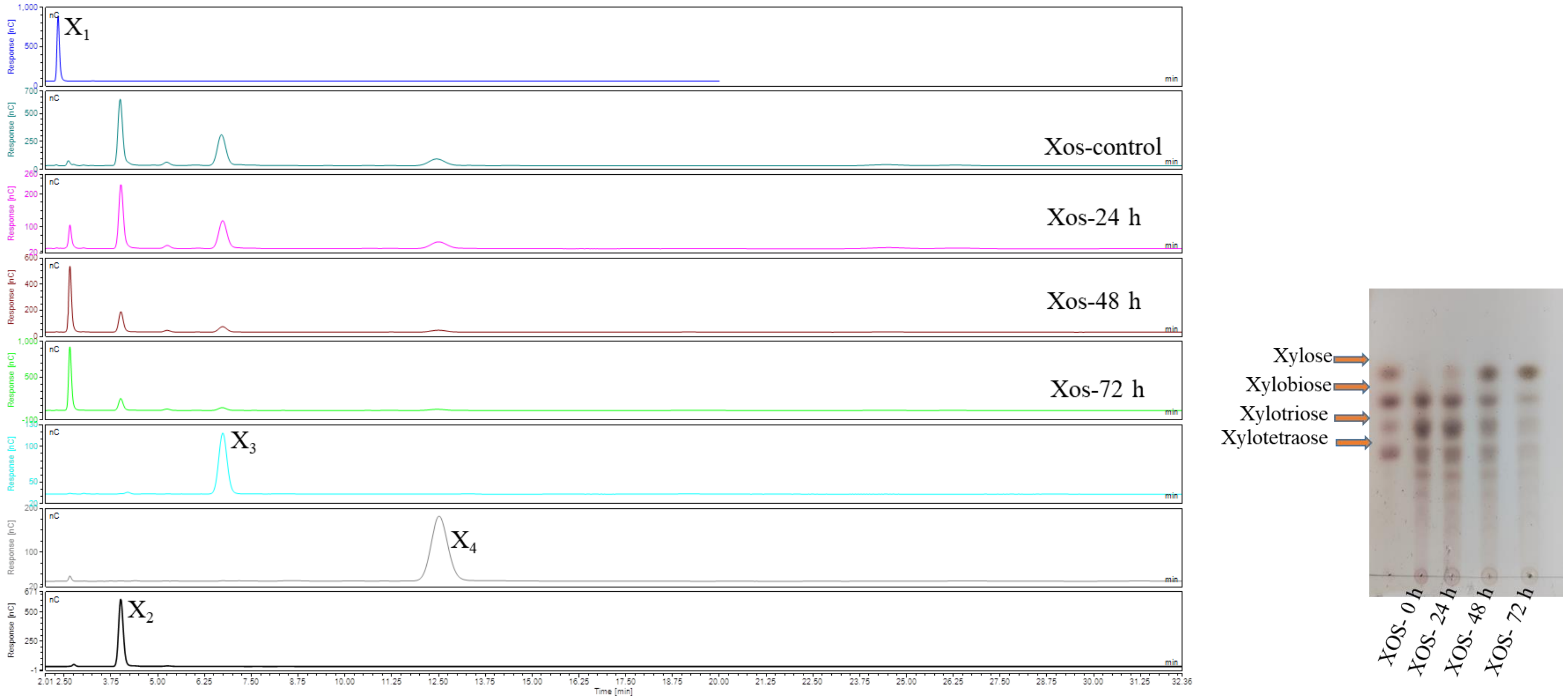

Figure S4: Analysis of XOS utilisation by *B. producta* ATCC 27340. HPAEC-ED (A) and thin layer chromatography (B) were used to monitor XOS degradation products over time. X_1- xylose, X_2- xylobiose, X_3- xylotriose and X_4- xylotetraose.

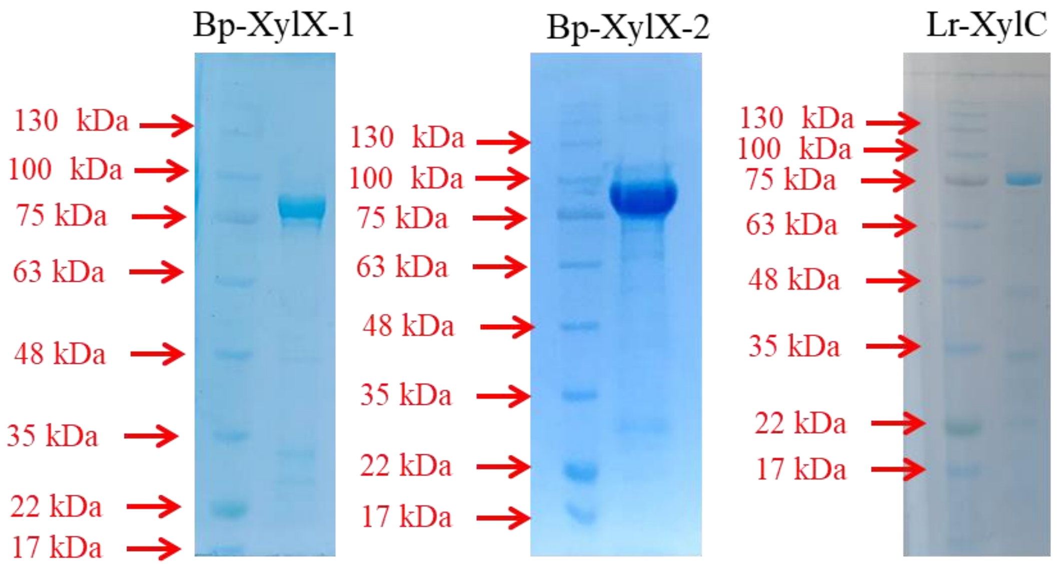

Figure S5A. Analysis of recombinant β-xylosidases on 10 % SDS-PAGE. Broad range (10-230 kDa) molecular weight marker was used. Apparent molecular weights are indicated with an arrow.

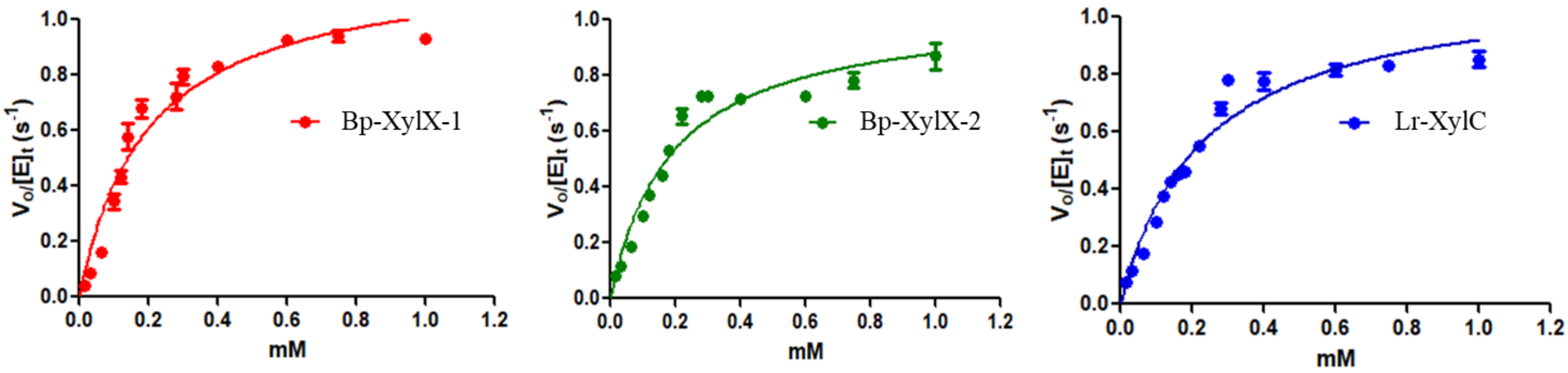

Figure S5B. Michaelis-Menten plots for Bp-XylX-1, Bp-XylX-2 and Lr-XylC using different concentrations of pNP-Xyl. All reactions were performed in triplicate. Data were plotted using GraphPad Prism (v 5.04).

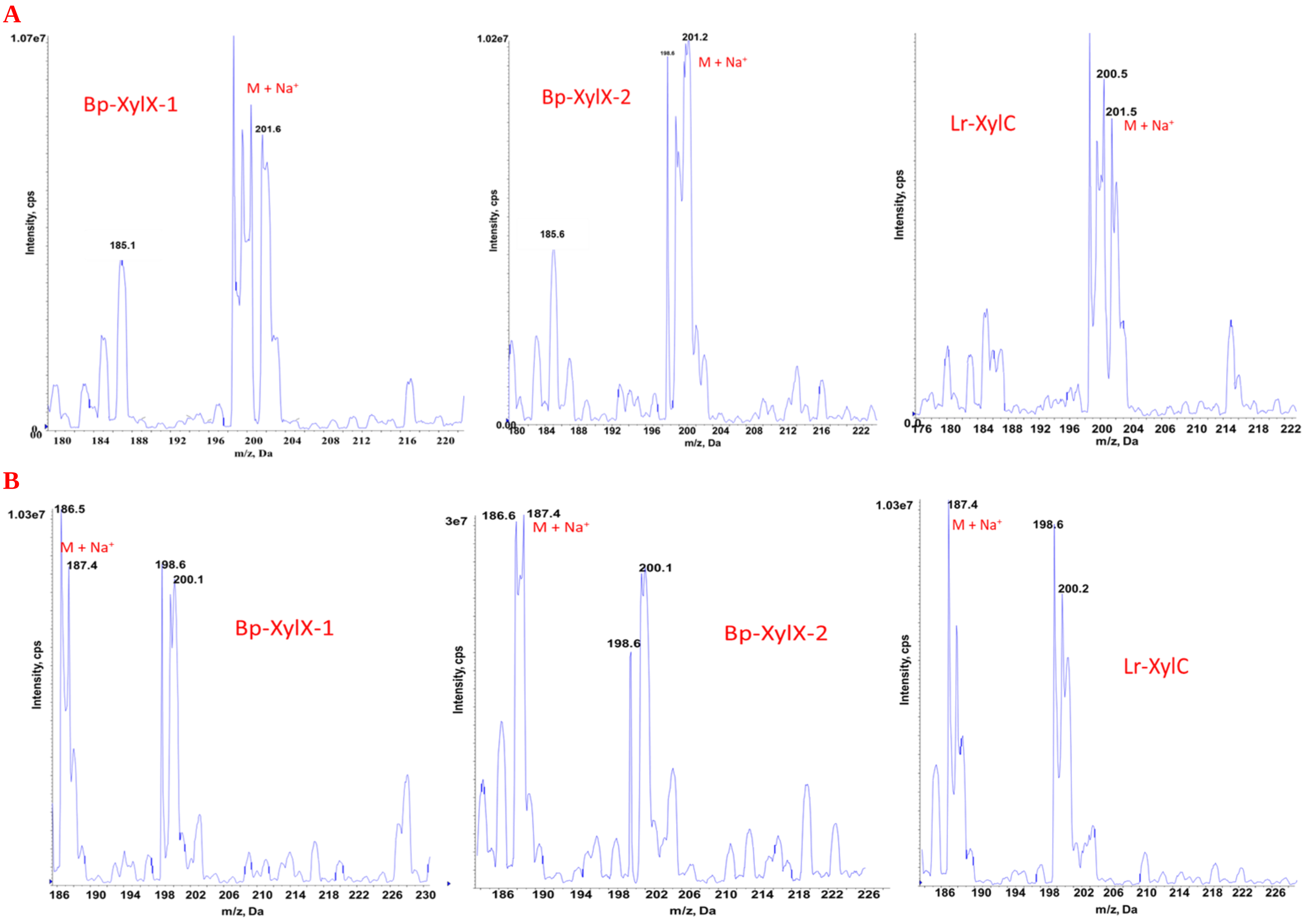

Figure S6: Transxylosylation activities of recombinant xylosidases. ESI-MS of ethoxylated (A) and methoxylated (B) xyloses catalysed by β-xylosidases (Bp-XylX-1, Bp-XylX-2 and Lr-XylC). *p*NP-Xyl was used as acceptor at 2 mM final concentration.

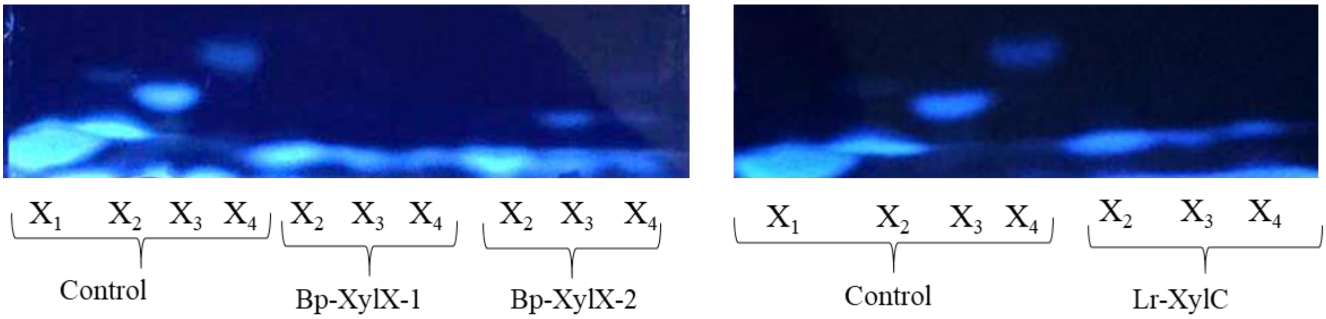

Figure S7: Enzymatic activity of recombinant β-xylosidases on xylobiose, xylotriose and xylotetraose. The products of the enzymatic reaction of Bp-XylX-1, Bp-XylX-2 and Lr-XylC on xylobiose, xylotriose and xylotetraose were analysed by FACE.

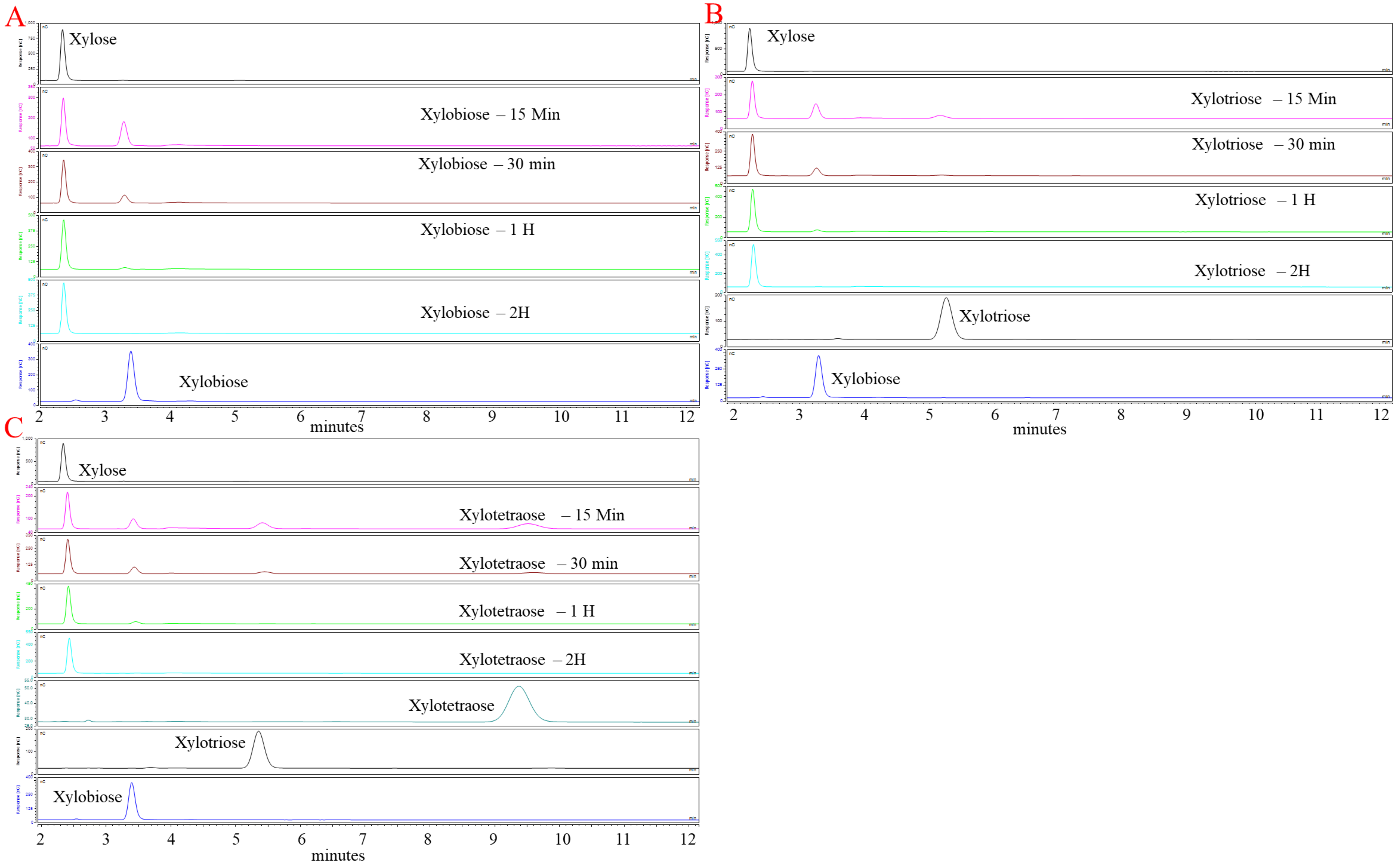

Figure S8: Digestion of xylobiose, xylotriose and xylotetraose by the Bp-XylX-1 from *B. producta* ATCC 27340. Digestion of xylobiose (A), xylotriose (B) and xylotetraose (C) was analysed by HPAEC-ED. Isocratic gradient of 200 mM NaOH was used.

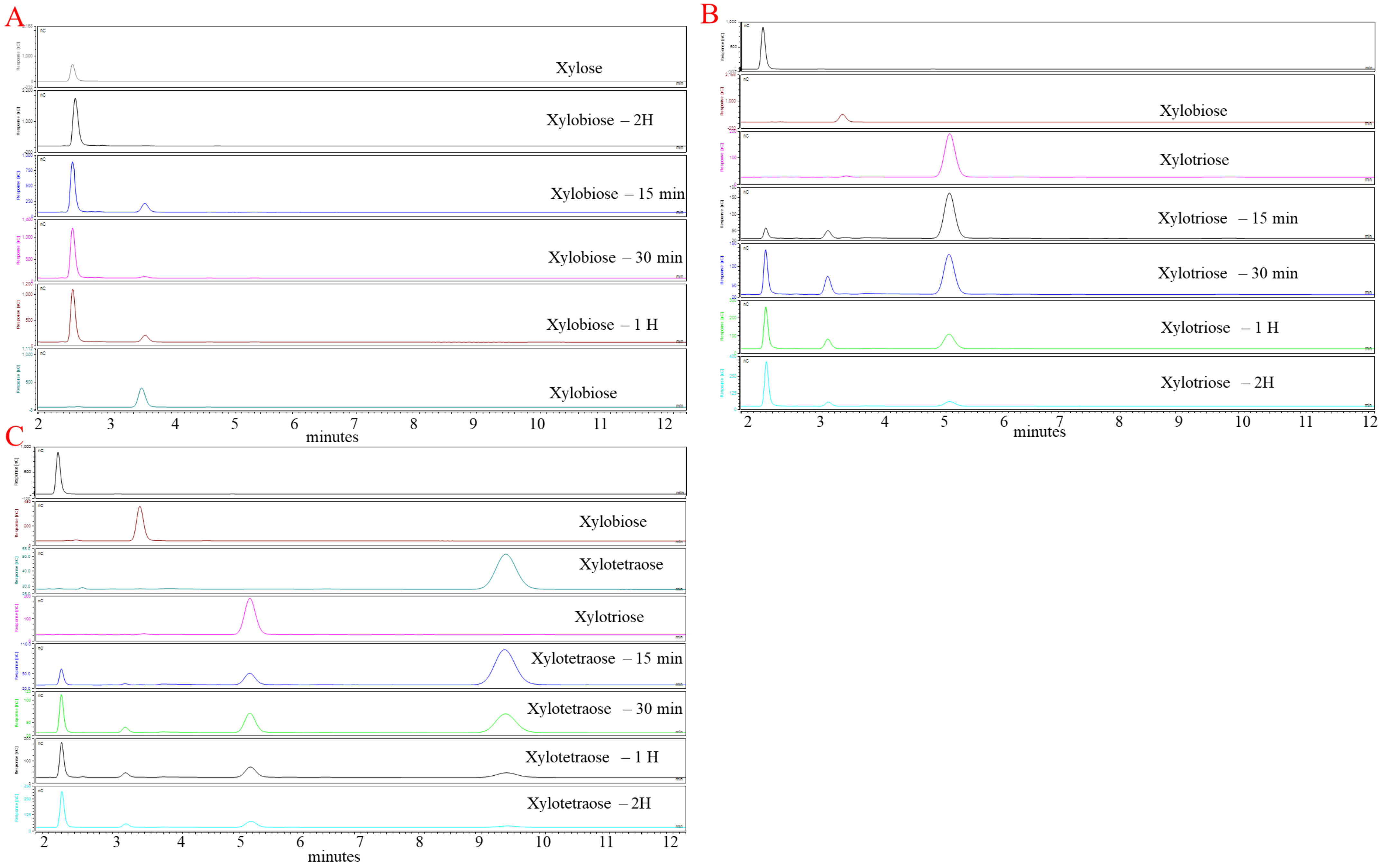

Figure S9: Digestion of xylobiose, xylotriose and xylotetraose by the Bp-XylX-2 from *B. producta* ATCC 27340. Digestion of xylobiose (A), xylotriose (B) and xylotetraose (C) was analysed by HPAEC-ED. Isocratic gradient of 200 mM NaOH was used.

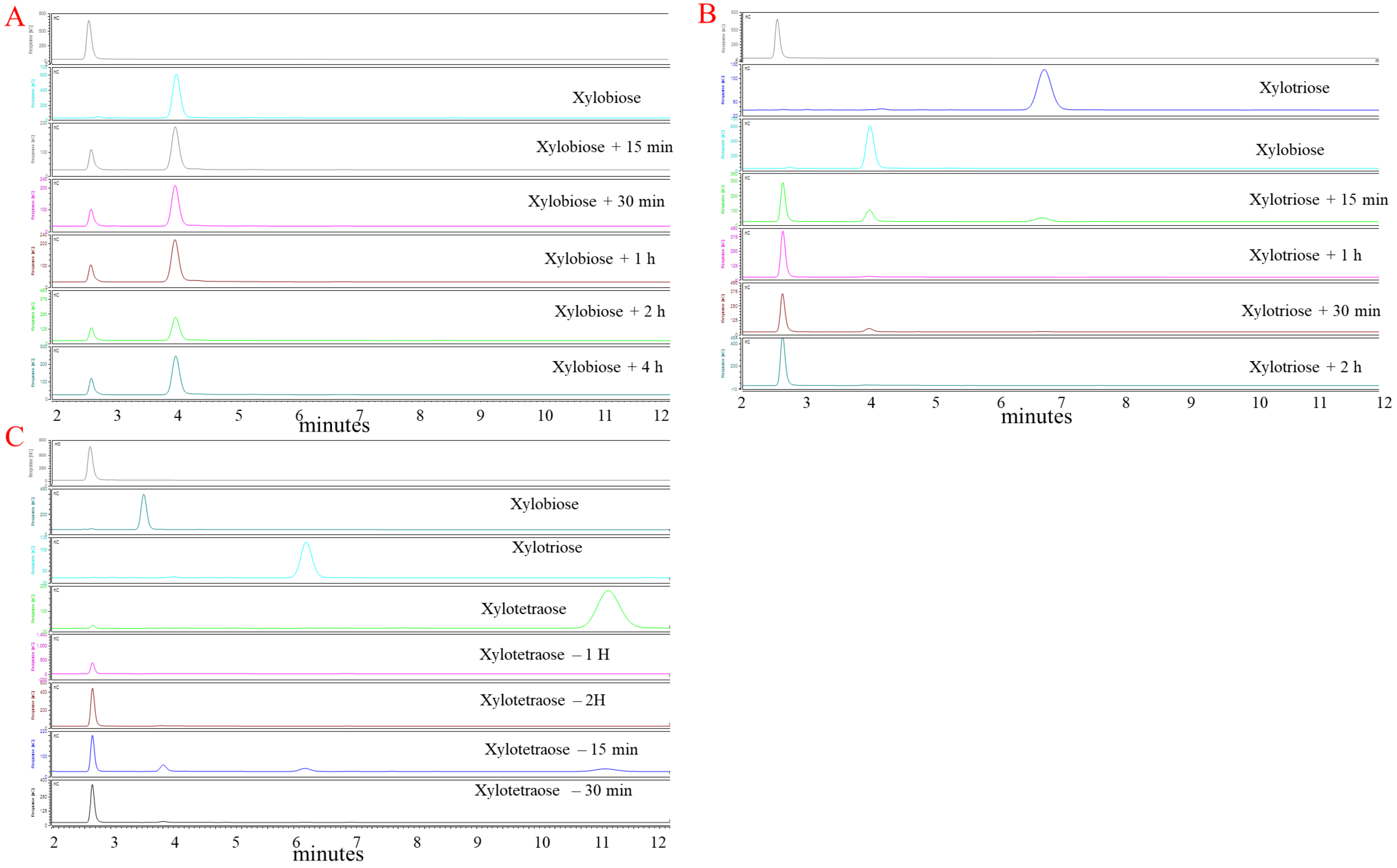

Figure S10: Digestion of xylobiose, xylotriose and xylotetraose by the Lr-XylC from *L. reuteri* ATCC53608. Digestion of xylobiose (A), xylotriose (B) and xylotetraose (C) was analysed by HPAEC-ED. Isocratic gradient of 150 mM NaOH.

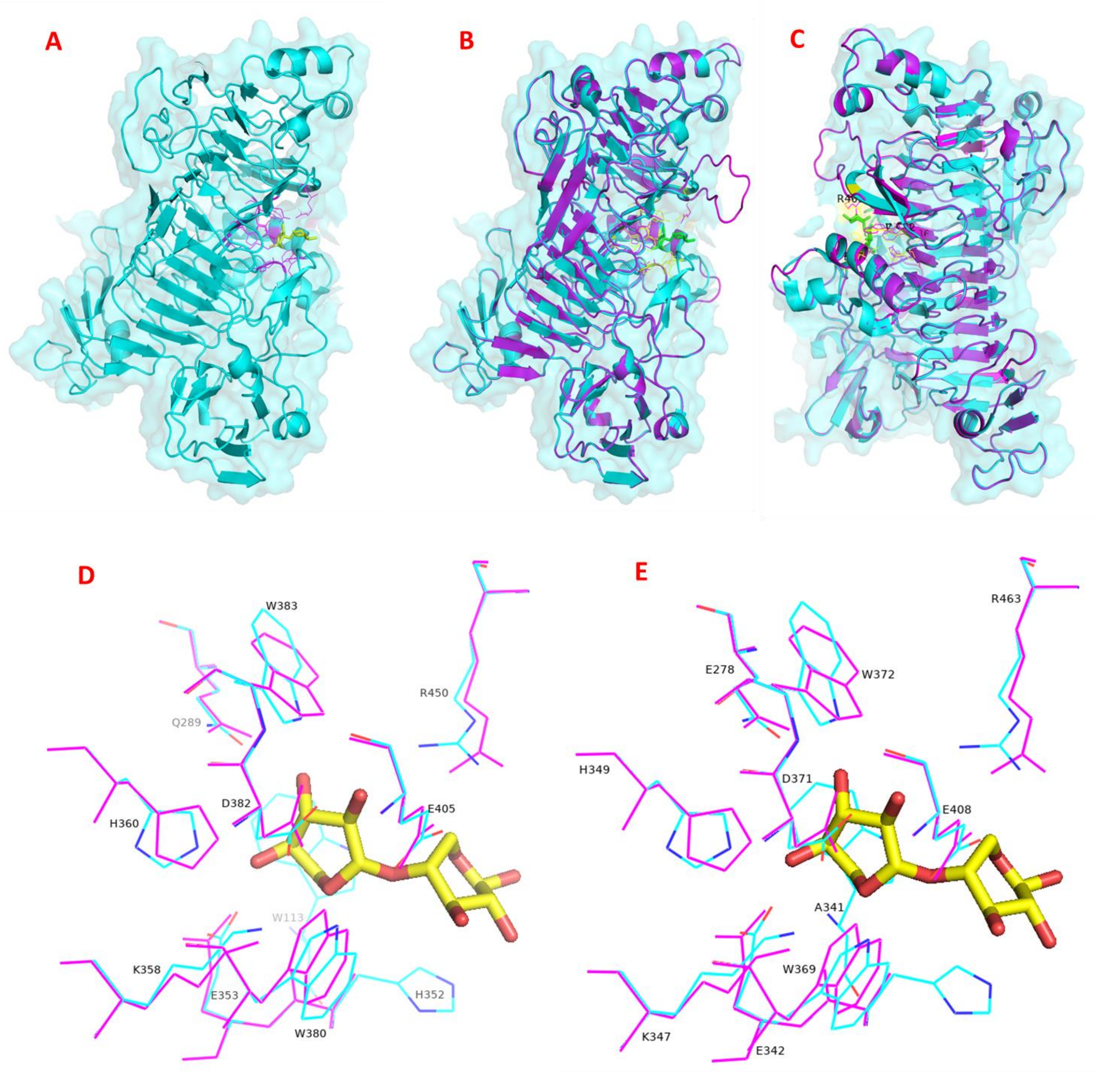

Figure S11: Homology modelling of Bp-XylX-1. Crystal structure (PDB ID: 3VSU) of Ts-XOS and it is represented in the transparent surface and cartoon representations (A). The Bp-XylX-1 (magenta) superimposed on Ts-XOS and showed in (B) rear view and (C) front view. Critical amino acid (AA) residues present in vicinity of xylobiose of Ts-XOS (cyan) and Bp-XylX-1 (magenta) at 5 Å (D and E). Labelled AA residues of Ts-XOS (D) and Bp-XylX-1 (E).

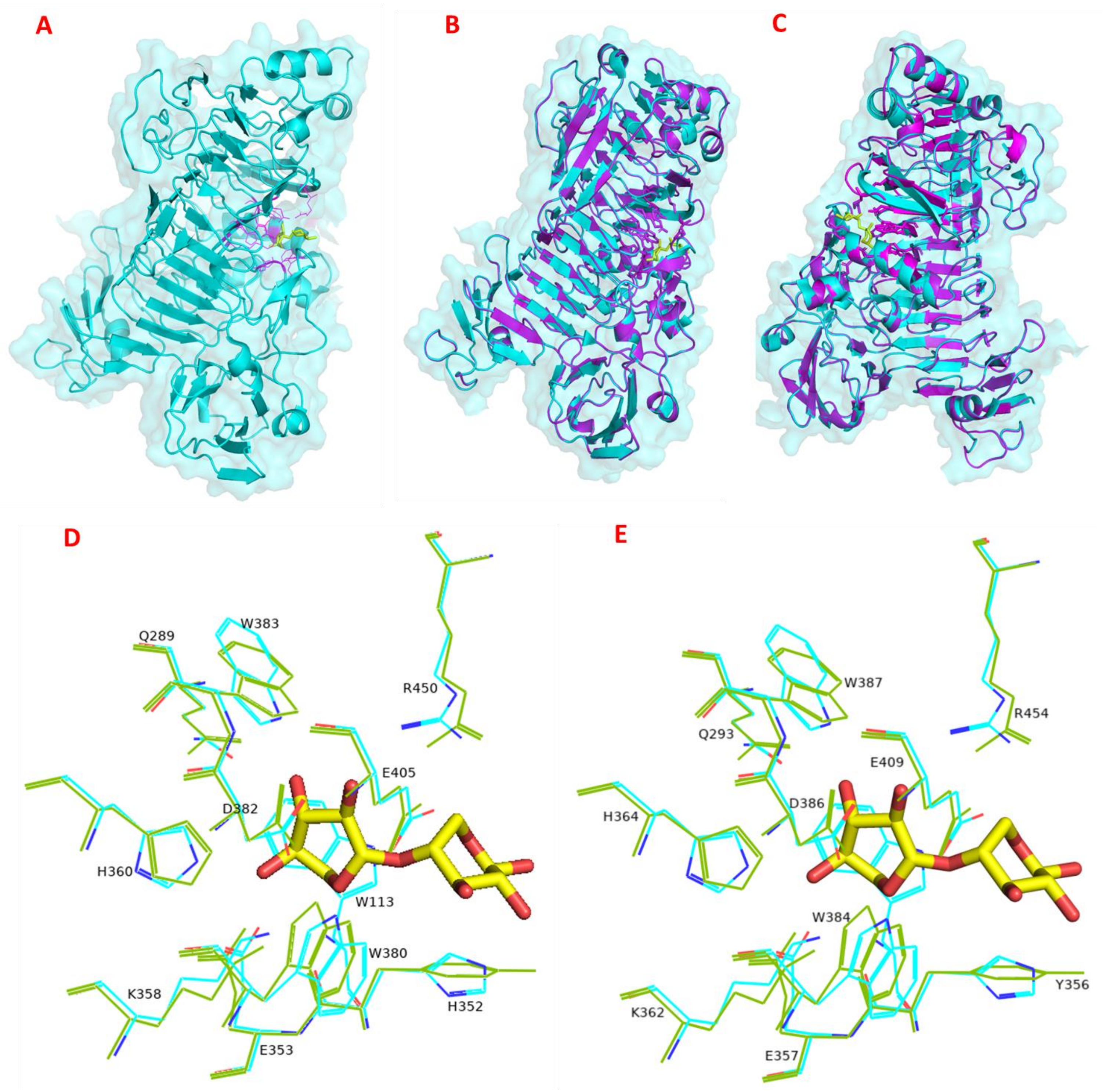

Figure S12: Homology modelling of Bp-XylX-2. Crystal structure (PDB ID: 3VSU) of Ts-XOS and it is represented in the transparent surface and cartoon representations (A). The Bp-XylX-2 (magenta) superimposed on Ts-XOS and showed in (B) rear view and (C) front view. Critical amino acid (AA) residues present in vicinity of xylobiose of Ts-XOS (cyan) and Bp-XylX -2 (green) at 5 Å (D and E). Labelled AA residues of Ts-XOS (D) and Bp-XylX-2 (E).

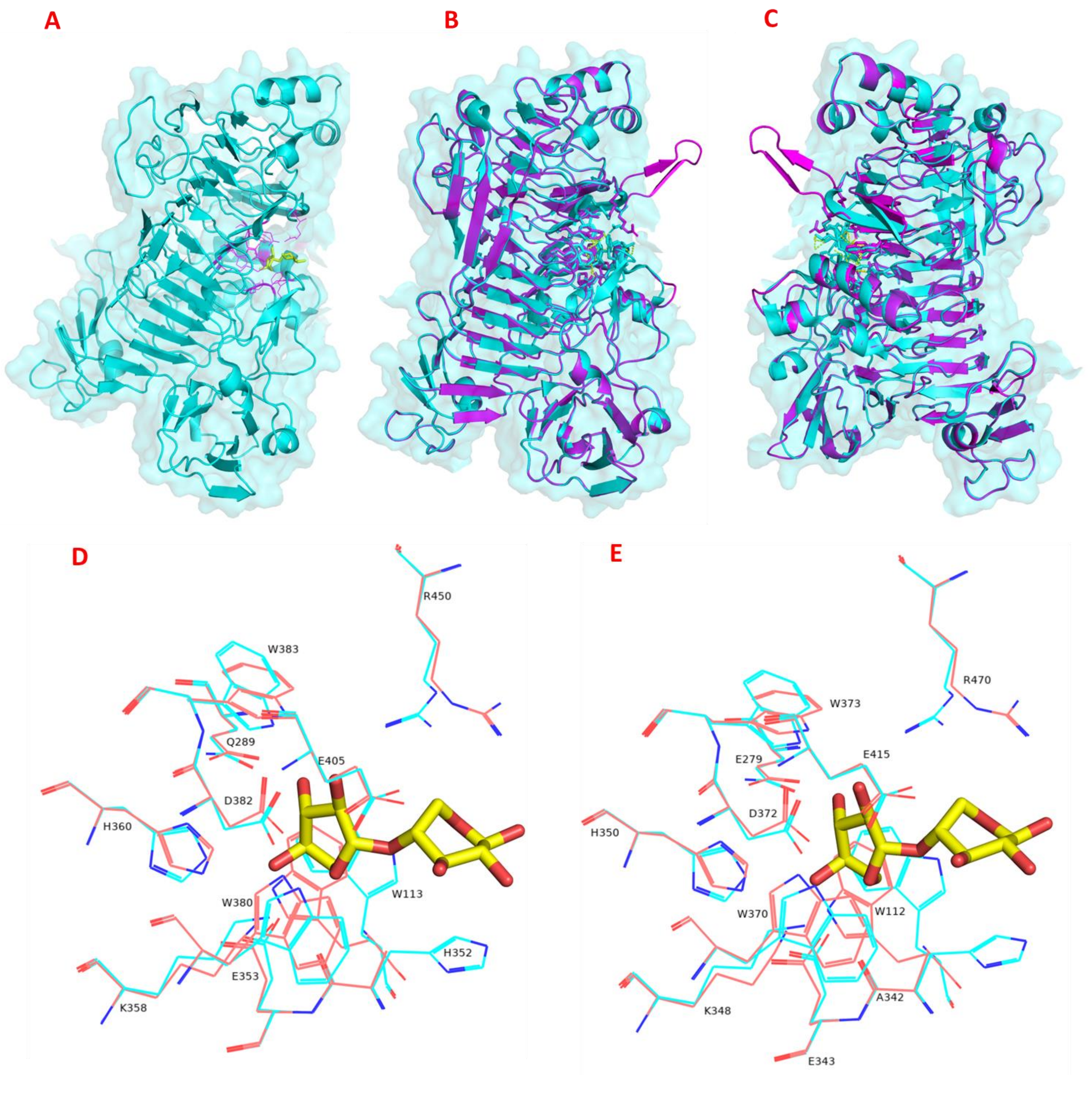

Figure S13: Homology modelling of Lr-XylC. Crystal structure (PDB ID: 3VSU) of Ts-XOS and it is represented in the transparent surface and cartoon representations (A). The Lr- XylC (magenta) superimposed on Ts-XOS and showed in (B) rear view and (C) front view. Critical amino acid (AA) residues present in vicinity of xylobiose of Ts-XOS (cyan) and Lr-XylC (red) at 5 Å (D and E). Labelled AA residues of Ts-XOS (D) and Lr-XylC (E).

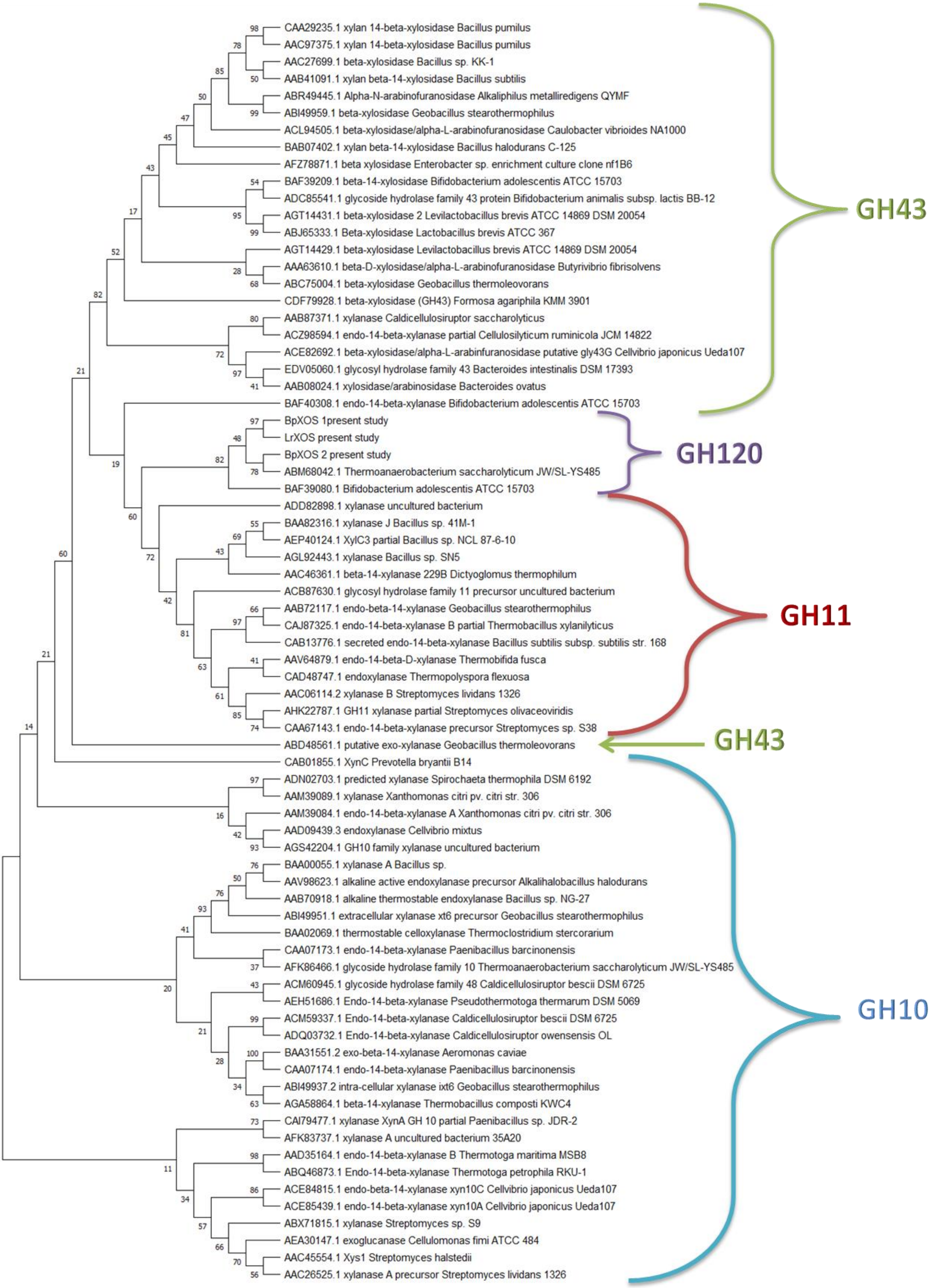

CAA29235.1 xylan 14-beta-xylosidase Bacillus pumilus
AAC97375.1 xylan 14-beta-xylosidase Bacillus pumilus
AAC27699.1 beta-xylosidase Bacillus sp. KK-1
AAB41091.1 xylan beta-14-xylosidase Bacillus subtilis
ABR49445.1 Alpha-N-arabinofuranosidase Alkaliphilus metalliredigens QYMF
ABI49959.1 beta-xylosidase Geobacillus stearothermophilus
ACL94505.1 beta-xylosidase/alpha-L-arabinofuranosidase Caulobacter vibrioides NA1000
BAB07402.1 xylan beta-14-xylosidase Bacillus halodurans C-125
AFZ78871.1 beta xylosidase Enterobacter sp. enrichment culture clone nf1B6
BAF39209.1 beta-14-xylosidase Bifidobacterium adolescentis ATCC 15703
ADC85541.1 glycoside hydrolase family 43 protein Bifidobacterium animalis subsp. lactis BB-12
AGT14431.1 beta-xylosidase 2 Levilactobacillus brevis ATCC 14869 DSM 20054
ABJ65333.1 Beta-xylosidase Lactobacillus brevis ATCC 367
AGT14429.1 beta-xylosidase Levilactobacillus brevis ATCC 14869 DSM 20054
AAA63610.1 beta-D-xylosidase/alpha-L-arabinofuranosidase Butyrivibrio fibrisolvens
ABC75004.1 beta-xylosidase Geobacillus thermoleovorans
CDF79928.1 beta-xylosidase (GH43) Formosa agariphila KMM 3901
AAB87371.1 xylanase Caldicellulosiruptor saccharolyticus
ACZ98594.1 endo-14-beta-xylanase partial Cellulosilyticum ruminicola JCM 14822
ACE82692.1 beta-xylosidase/alpha-L-arabinfuranosidase putative gly43G Cellvibrio japonicus Ueda107
EDV05060.1 glycosyl hydrolase family 43 Bacteroides intestinalis DSM 17393
AAB08024.1 xylosidase/arabinosidase Bacteroides ovatus
BAF40308.1 endo-14-beta-xylanase Bifidobacterium adolescentis ATCC 15703
BpXOS 1present study
LrXOS present study
BpXOS 2 present study
ABM68042.1 Thermoanaerobacterium saccharolyticum JW/SL-YS485
BAF39080.1 Bifidobacterium adolescentis ATCC 15703
ADD82898.1 xylanase uncultured bacterium
BAA82316.1 xylanase J Bacillus sp. 41M-1
AEP40124.1 XylC3 partial Bacillus sp. NCL 87-6-10
AGL92443.1 xylanase Bacillus sp. SN5
AAC46361.1 beta-14-xylanase 229B Dictyoglomus thermophilum
ACB87630.1 glycosyl hydrolase family 11 precursor uncultured bacterium
AAB72117.1 endo-beta-14-xylanase Geobacillus stearothermophilus
CAJ87325.1 endo-14-beta-xylanase B partial Thermobacillus xylanilyticus
CAB13776.1 secreted endo-14-beta-xylanase Bacillus subtilis subsp. subtilis str. 168
AAV64879.1 endo-14-beta-D-xylanase Thermobifida fusca
CAD48747.1 endoxylanase Thermopolyspora flexuosa
AAC06114.2 xylanase B Streptomyces lividans 1326
AHK22787.1 GH11 xylanase partial Streptomyces olivaceoviridis
CAA67143.1 endo-14-beta-xylanase precursor Streptomyces sp. S38
ABD48561.1 putative exo-xylanase Geobacillus thermoleovorans
CAB01855.1 XynC Prevotella bryantii B14
ADN02703.1 predicted xylanase Spirochaeta thermophila DSM 6192
AAM39089.1 xylanase Xanthomonas citri pv. citri str. 306
AAM39084.1 endo-14-beta-xylanase A Xanthomonas citri pv. citri str. 306
AAD09439.3 endoxylanase Cellvibrio mixtus
AGS42204.1 GH10 family xylanase uncultured bacterium
BAA00055.1 xylanase A Bacillus sp.
AAV98623.1 alkaline active endoxylanase precursor Alkalihalobacillus halodurans
AAB70918.1 alkaline thermostable endoxylanase Bacillus sp. NG-27
ABI49951.1 extracellular xylanase xt6 precursor Geobacillus stearothermophilus
BAA02069.1 thermostable celloxylanase Thermoclostridium stercorarium
CAA07173.1 endo-14-beta-xylanase Paenibacillus barcinonensis
AFK86466.1 glycoside hydrolase family 10 Thermoanaerobacterium saccharolyticum JW/SL-YS485
ACM60945.1 glycoside hydrolase family 48 Caldicellulosiruptor bescii DSM 6725
AEH51686.1 Endo-14-beta-xylanase Pseudothermotoga thermarum DSM 5069
ACM59337.1 Endo-14-beta-xylanase Caldicellulosiruptor bescii DSM 6725
ADQ03732.1 Endo-14-beta-xylanase Caldicellulosiruptor owensensis OL
BAA31551.2 exo-beta-14-xylanase Aeromonas caviae
CAA07174.1 endo-14-beta-xylanase Paenibacillus barcinonensis
ABI49937.2 intra-cellular xylanase ixt6 Geobacillus stearothermophilus
AGA58864.1 beta-14-xylanase Thermobacillus composti KWC4
CAI79477.1 xylanase XynA GH 10 partial Paenibacillus sp. JDR-2
AFK83737.1 xylanase A uncultured bacterium 35A20
AAD35164.1 endo-14-beta-xylanase B Thermotoga maritima MSB8
ABQ46873.1 Endo-14-beta-xylanase Thermotoga petrophila RKU-1
ACE84815.1 endo-beta-14-xylanase xyn10C Cellvibrio japonicus Ueda107
ACE85439.1 endo-14-beta-xylanase xyn10A Cellvibrio japonicus Ueda107
ABX71815.1 xylanase Streptomyces sp. S9
AEA30147.1 exoglucanase Cellulomonas fimi ATCC 484
AAC45554.1 Xys1 Streptomyces halstedii
AAC26525.1 xylanase A precursor Streptomyces lividans 1326
GH43
GH120
GH11
GH43
GH10

Figure S14: Evolutionary analysis by Maximum Likelihood method. The evolutionary history of different enzymes belonging to glycoside hydrolase family 10, 11, 43 and 120 were conducted with the maximum likelihood method and Jones-Taylor-Thornton matrix-based model (Jones, *et al.*, 1992). The evolutionary history of the taxa is represented with the bootstrap value which is inferred from 500 replicates (Felsenstein, 1985). The value of bootstrap test is shown next to branches (Felsenstein, 1985). Evolutionary analyses were conducted in MEGA-x (Kumar, *et al.*, 2018).

Table: S1-A list of primer used for cloning of genes in this study

**Name of primer**	**Sequence**
Bp- XylX -1-F	ACGCCATATGATGATTTATTATGTAGCCGCA
Bp- XylX -1-R	TTAGCTCGAGTTATAATACGTTCCCGGATT
Bp- XylX -2-F	TGTAGCTAGCATGAACAGAGAATACCATGT
Bp- XylX -2-R	TTTACTCGAGTTATCCCCAGACTTTTATTT
LR- XylC -F	AACAGCATATGATGAATATTTATGTAGACAA
LR- XylC -R	GTTCACTCGAGTTAAAATAGTCTACTACTTG
Bp- XylX *t*-F	AAAGGCTAGCATGTGGCATCAAAGTGGAAA
Bp- XylX *t*-R	CAAGCTCGAGTCAGCCCGCGTTAAGGGAAC

Underlines are indicating cleavage site of restriction enzymes. Xyl*t*: t- transporter.

Table: S2-A list of primer used for site directed mutagenesis

**Name of primer**	**Sequence**
Bp- XylX 1-D371_F	TCTGTGGCTTgcaTGGCAGGC
Bp- XylX 1-D371_R	CCACGGGTACAGTGATGGAAATGGTTG
Bp- XylX 1-E408_F	TATCTTTGTTgcaGTTTCCCACGGC
Bp- XylX 1-E408_R	TCCTCTGCGAGAGCCAGACC
Bp- XylX 2-D386_F	CACCTGGCTTgcaTGGCAGGC
Bp- XylX 2-D386_R	CCAAGGGTACAGTCATGGATATTATTGTTATGGATCTGTAC
Bp- XylX 2-E409_F	CCTGATGGTTgcaGTGACACATG
Bp- XylX 2-E409_R	TCACGGTCATTGTTGTAGTAGAGGT
LR_ XylC -D372_F	CTTATGGTTGgcaTGGCAGGCT
LR_ XylC -D372_R	CCACGAGTACAATGATGAATATGATTATGACGATATG
LR_ XylC -E415_F	TATGTGGATTgcaGTTTCTCATGGG
LR_ XylC -E415_R	TCTTCCCCGAGACCAGATAAAACA

Small letters indicate mutation site.

Table S3: Expression of CAZymes related proteins when these bacteria were grown on xylo-oligosaccharides.

**Identified proteins**	**Accession number**	**MW (kDa)**	***L. reuteri* 53608**	***L. reuteri* 100-23C**	**Signal peptide (Sec/SPI)**	**Homologous similarity to *L. reuteri* 53608 in *L. reuteri* 100-23C (%)**	**Homologous similarity to L. reuteri 53608 in *B. producta* ATCC 27340 (%)**
Xylose isomerase	A0A073JNR5_LACRE	51	47	39	No	99	ND
Putative xylosidase	A0A0S4NRU4_LACRE	77	51	44	No	98	XOS-1 (59%) and XOS-2 (40%)
Maltose epimerase	A0A143Q1I0_LACRE	38	17	17	No	98	31
Glucohydrolase	A0A0S4NKN9_LACRE	66	2	2	No	98	oligo-1,6-glucosidase (57%)
L-arabinose isomerase	A0A098QZE8_LACRE	54	26	26	No	100	48
Maltose phosphorylase	A0A143Q1E1_LACRE	87	42	42	No	99	33
Xylulokinase	A0A073K0P2_LACRE	56	24	23	No	100	42
Galactokinase	A0A0S4NN99_LACRE	44	20	16	No	99	45
β-Galactosidase	A0A143Q191_LACRE	74	19	19	No	98	49
α-Glucosidase	A0A143PYJ9_LACRE	88	8	33	No	97	33
α-Galactosidase	A0A0S4NP55_LACRE	84	29	10	No	99	46
β -Galactosidase	A0A0S4NQK4_LACRE	35	11	10	No	98	34
MFS transporter	A0A0S4NND3_LACRE	34	19	7	No	98	45
α / β hydrolase	A0A143Q035_LACRE	28	9	9	No	97	ND
Sucrose phosphorylase	Q1KMT7_LACRE	56	6	6	No	99	27
Xylose isomerase	A0A0S4NL92_LACRE	32	4	5	No	98	ND
MFS transporter	A0A0S4NNZ2_LACRE	51	4	4	No	99	ND
α / β - hydrolase	A0A0S4NN34_LACRE	31	5	3	Yes	99	ND
Putative xylose repressor (ROK family protein)	A0A0S4NPC3_LACRE	45	5	4	No	99	29
Glycoside-Pentoside-Hexuronide family cation symporter	A0A0S4NL78_LACRE	71	5	5	No	99	28
ABC transporter permease	A0A0S4NPD1_LACRE	55	5	0	Yes	99	43
Maltose O-acetyltransferase	A0A0S4NP60_LACRE	21	5	0	No	97	42

Signal peptide and homologous similarity between proteins were determined using SignalP-5.0 and the Integrated Microbial Genomes (IMG) system server respectively. ND-not-detected. In case of multiple homologous similarities, only highest shown sequence similarity was mentioned in the table. Total numbers of peptide counts are mentioned in the table.

References

Felsenstein J (1985) Confidence limits on phylogenies: An approach using the bootstrap. *Evolution* **39**: 783-791.

Tyanova S, Temu T & Cox J (2016) The MaxQuant computational platform for mass spectrometry-based shotgun proteomics. *Nat Protoc* **11**: 2301-2319.

Wessel D & Flugge UI (1984) A method for the quantitative recovery of protein in dilute solution in the presence of detergents and lipids. *Anal Biochem* **138**: 141-143.
